# Eccentricity and obliquity paced carbon cycling in the Early Triassic and implications for post-extinction ecosystem recovery

**DOI:** 10.1038/srep27793

**Published:** 2016-06-13

**Authors:** Wanlu Fu, Da-yong Jiang, Isabel P. Montañez, Stephen R. Meyers, Ryosuke Motani, Andrea Tintori

**Affiliations:** 1 Laboratory of Orogenic Belt and Crustal Evolution, Ministry of Education; Department of Geology and Geological Museum, Peking University, Yiheyuan Street. 5, Beijing 100871, People’s Republic of China; 2 Department of Earth and Planetary Sciences, University of California, Davis, One Shields Avenue, Davis, California 95616, USA; 3 Department of Geoscience, University of Wisconsin, 1215 West Dayton St, Madison, WI 53076, USA; 4 Dipartimento di Scienze della Terra, Università degli Studi di Milano, Via Mangiagalli 34-20133 Milano, Italy

## Abstract

The timing of marine ecosystem recovery following the End Permian Mass Extinction (EPME) remains poorly constrained given the lack of radiometric ages. Here we develop a high-resolution carbonate carbon isotope (δ^13^C_carb_) record for 3.20 million years of the Olenekian in South China that defines the astronomical time-scale for the critical interval of major evolutionary and oceanic events in the Spathian. δ^13^C_carb_ documents eccentricity modulation of carbon cycling through the period and a strong obliquity signal. A shift in phasing between short and long eccentricity modulation, and amplification of obliquity, is nearly coincident with a 2% decrease in seawater δ^13^C_DIC,_ the last of a longer-term stepped decrease through the Spathian. The mid-Spathian shift in seawater δ^13^C_DIC_ to typical thermocline values is interpreted to record a major oceanic reorganization with global climate amelioration. Coincidence of the phasing shift with the first occurrence of marine reptiles (248.81 Ma), suggests that their invasion into the sea and the onset of a complex ecosystem were facilitated by restoration of deep ocean ventilation linked mechanistically to a change in the response of the oceanic carbon reservoir to astronomical forcing. Together these records place the first constraints on the duration of the post-extinction recovery to 3.35 myr.

Previous studies have shown that post-extinction recovery from the EPME was delayed^1^,^2^,^3^,^4^ until Middle Triassic due to anomalous oceanic conditions during the Early Triassic, elevated CO_2_ and surface temperatures^5^,^6^,^7^, widespread anoxia^8^,^9^,^10^,^11^,^12^,^13^ and high primary productivity^14^,^15^. However, discovery of an Early Triassic (Griesbachian to Early Smithian) rapid recovery of benthic faunas including bivalves, gastropods, brachiopods, and ostracods in Oman and the South China Block argues against persistent widespread anoxia^16^,^17^ and challenge the delayed-recovery hypothesis. Rather, a later period of widespread benthic anoxia and hot tropical temperatures at the Smithian-Spathian boundary created a new biocrisis with slowed rate of recovery^6^,^7^, making the pattern of post-extinction recovery more complex. Notably, most prior studies of the biotic recovery have focused on invertebrate benthic marine faunas^2^,^3^,^4^,^16^,^17^. Mesozoic marine reptiles, such as ichthyopterygians and sauropterygians, first occurred in the Early Triassic as a part of the transition from the ‘Paleozoic Fauna’ to the ‘Modern Fauna’, and dominated the Mesozoic seas^18^,^19^. Our collection of more than 80 well-preserved and multi-clade mid-Spathian marine reptile specimens from the Majiashan Section, Chaohu, South China documents that predatory marine tetrapods diversified in the Early Triassic and were well adapted to life in the sea, indicating a habitable marine environment in the mid-Spathian at Chaohu, which is earlier then previously suggested. The diverse predators, including fish and marine reptiles, and abundant invertebrates such as bivalves, ammonoids and arthropods, form the Chaohu Fauna, represent a nearly complete ecosystem. The first appearance of abundant and multi-clade Mesozoic marine reptiles in Chaohu provides the first glimpse of recovering biotic structure reaching a high trophic level. The lack of geochronologic data, however, has precluded constraining the age of the Chaohu Fauna and thus the timing of post-extinction recovery remains poorly known.

Deep-sea δ^13^C_carb_ records of mid-Cretaceous to Cenozoic age reveal a persistent long-eccentricity (405-kyr) cyclicity, which records astronomical pacing of carbon cycling among Earth’s surface reservoirs^20^,^21^,^22^,^23^,^24^. Astronomical cyclicity is also recognized following the EPME^25^,^26^,^27^, when potentially the largest carbon cycle perturbation of the Phanerozoic was followed by a series of disturbances throughout the Early Triassic interval of protracted marine ecosystem recovery^2^,^28^. Astronomical cycles in carbon cycling, however, have not yet been recognized leaving ambiguous the timing of the post-extinction recovery and the role of astronomical pacing of climate and carbon cycling through a significant portion of the biotic recovery phase. Furthermore, constraining the drivers of major carbon cycle perturbation is fundamental to understanding climate and biosphere dynamics throughout Earth history^29^,^30^.

The nature of the mechanistic link between post-extinction carbon cycle perturbations and the Early Triassic biotic recovery has been the focus of much research invoking wholesale changes in ocean oxygenation and water column stratification^13^,^15^. Major oceanic restructuring is inferred to have occurred in response to global warming, likely driven by episodic release of exogenic carbon from Siberian Trap volcanism^6^,^31^. Conversely, the role of internal mechanisms and feedbacks in the Earth’s climate system, including cycling through the surface carbon reservoirs, has not been investigated in the context of the recovery of life after the largest extinction since the evolution of metazoans. In this study, we develop a new high-resolution C isotopic record of seawater dissolved inorganic carbon (δ^13^C_DIC_) for the end-Smithian through Spathian, utilize it to construct an astrononmical time scale, and in turn, apply it to infer major changes in ocean structure mechanistically linked to the timing of ecosystem recovery.

## Results

**Figure 1:**
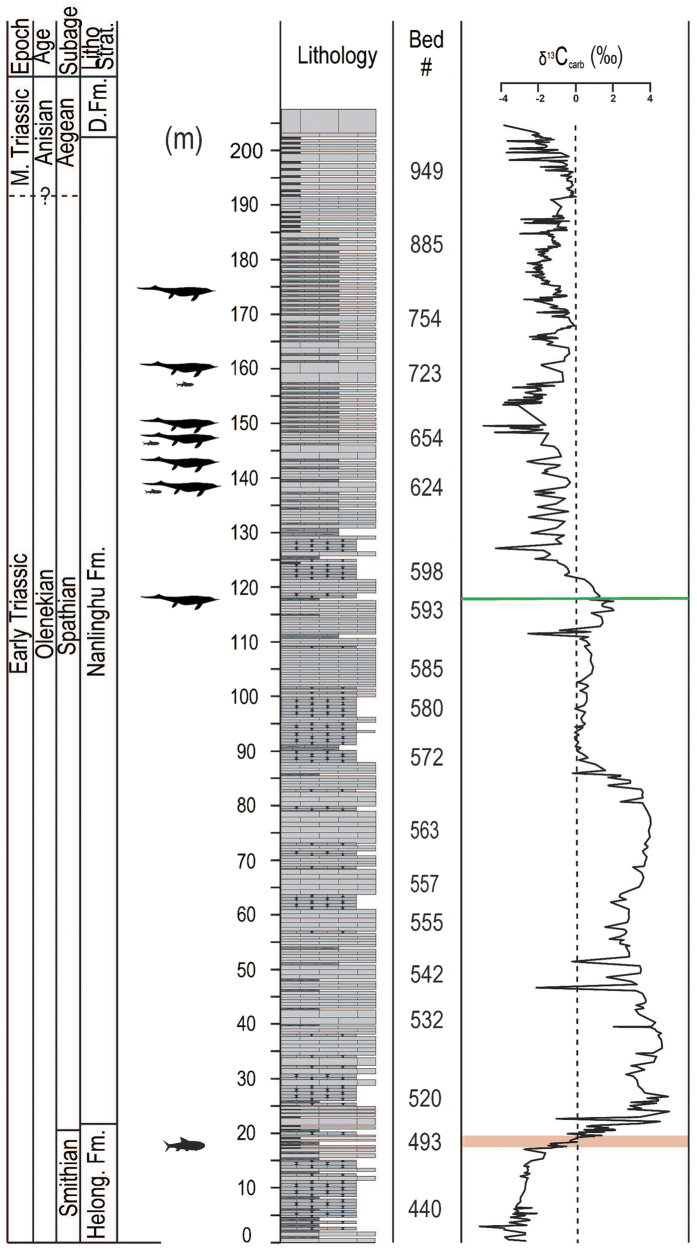
Stratigraphy and δ^13^C_carb_ data values on column for Majiashan section (see Supplementary Table S1) with vertebrate fossil distribution. The pink horizontal line shows the stratigraphic level of the end-Smithian biocrisis, coincident with a rapid increase in δ^13^C_carb_; the green horizontal line shows the first occurrence of Triassic marine reptiles. Helong. Fm.: Helongshan Formation; D. Fm.: Dongma’anshan Formation. The large silhouettes to the left of the lithology are marine reptiles; the small silhouettes to the left of the lithology (and one medium silhouette) are fish.

A high-resolution δ^13^C_carb_ record is constructed spanning the end-Smithian through Spathian (~250.55 to 247.32 Ma, Olenekian, Early Triassic)^32^,^33^, from an intermediate- to deep-water 200 to 500 m) slope succession in Majiashan, Chaohu, South China (Fig. 1). Observed fluctuations in δ^13^C_carb_ are interpreted to record secular changes in seawater δ^13^C_DIC_, an inference that is supported by the lack of correlation between δ^13^C_carb_ and lithofacies changes, δ^18^O_carb_, δ^13^C_org_ or wt.% CaCO_3_ (see Supplementary Figs S4 and S5). The veracity of the new δ^13^C_carb_ record is corroborated by similarity in long-term δ^13^C_carb_ trends between the Majiashan section and other global time-equivalent δ^13^C_carb_ records from South China and elsewhere^2^,^34^,^35^. The Majiashan δ^13^C_carb_ time-series (Fig. 1) reveals 3 scales of variation: (i) an abrupt 4% positive shift across the Smithian-Spathian boundary (S-S boundary) observed in previous records^31^, (ii) a subsequent 10^6^-yr-scale stepwise decrease from peak values of +2% to +4% in the early Spathian to minimum values of 0% to −3% in the middle-late Spathian; and (iii) low-amplitude (0.5–1%), shorter-term (10^5^-kyr scale) oscillations superimposed on the middle-late Spathian portion of the longer-term trend (see Supplementary Fig. S6).

**Figure 2:**
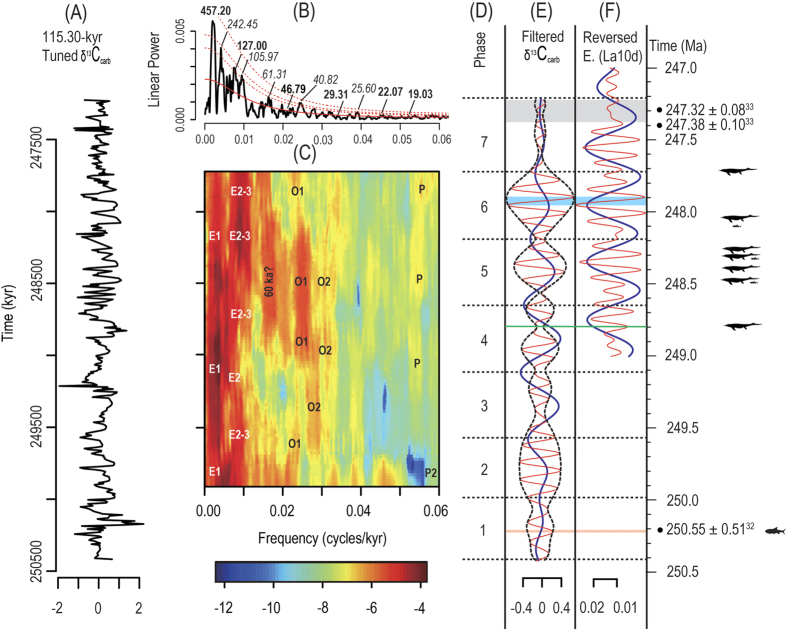
Spectra analysis results of the Majiashan δ^13^C_carb_ data following astronomical tuning and anchoring. (**A**) 115.30-kyr-tuned time series (see Supplementary Table S4). (**B**) Multi-taper method spectral analysis (MTM) results, using three tapers and a time-bandwidth product of 2p. The calibrated periods of significant peaks are shown in kyr; these peaks achieve the 90% confidence level for both the MTM harmonic F-test and the AR1 red noise model (bold) or the AR1 noise model only (italics) (see Supplementary Fig. S11, Supplementary Table S5). (**C**) Evolutive power spectral analysis (EPSA) results, using a moving window of 1000 kyr, 3 tapers and a time-bandwidth product of 2p (see Supplementary Fig. S12). A linear trend was removed from each window prior to the EPSA. Letters E1-P mark the eccentricity, obliquity and precession cycles separately (see Supplementary Table S2). (**D**) Phases that are defined by the minima of the filtered 405-kyr components (in **E**). Horizontal dashed lines are phase boundaries. (**E**) Filtered δ^13^C_carb_ time series for Majiashan section. Red and blue curves indicate short and long eccentricity cycles defined using bandpass filters of 0.0015–0.0028 cycles/m and 0.006–0.011 cycles/m, respectively. Black dotted lines show the instantaneous amplitude envelope of the δ^13^C_carb_ short eccentricity signal. (**F**) Theoretical eccentricity from La10d from 249.0 Ma-247.0 Ma. Red and blue curves indicate short and long eccentricity cycles. Note that La10d eccentricity curves are reversed to illustrate relationship between δ^13^C_carb_ maxima and eccentricity minima in Phase 6 marked by blue shading (see Supplementary Table S4). Radioisotopic dates from ref. 32,33. Correlation of latest Spathian interval constrained by radioisotopic ages shown by grey shading. The large silhouettes to the right of Panel F are marine reptiles; the small silhouettes to the right of Panel F (and one medium silhouette) are fish.

To evaluate potential astronomical cycles preserved in the record and their uncertainties, evolutive average spectral misfit (e-ASM) analysis^36^ was performed on the δ^13^C_carb_ record following data preparation (see R_analysis file) by statistically comparing the observed oscillations with theoretical periods from the astronomical models La04^37^ and La10d^38^ (see Supplementary Fig. S8, Supplementary Table S2). The results show continuous short-term eccentricity, obliquity and precession over a range of sedimentation rates (see Supplementary Fig. S8, Supplementary Table S3). Using the ASM-calibrated astronomical cycles, frequency domain minimal tuning was applied to generate a “floating” ATS (see Supplementary Fig. S10). Following frequency-domain minimal tuning using the average eccentricity period of 115.30 kyr (E2/2 + E3/2 from Supplementary Table S2; see Supplementary Fig. S10), the Majiashan δ^13^C_carb_ record exhibits power spectra peaks at periods of 457.20 kyr, 127.00 kyr, 46.79 kyr, 29.31 kyr, 22.07 kyr and 19.03 kyr (Fig. 2B, see Supplementary Fig. S11, Supplementary Table S5). A strong and continuous obliquity signal in the Evolutive Power Spectral Analysis (EPSA) of the tuned data (Fig. 2C, Supplementary Fig. S12), as well as a relatively persistent precession signal (Fig. 2C), provide independent confirmation of the short eccentricity minimal tuning implemented here.

The astrochronologic testing results provide a good basis for developing a nominal anchored astronomical time scale (ATS) and for dating the age of the first occurrence of Mesozoic marine reptiles, which invaded the marine environment and represent the start of the establishment of the new marine ecosystem dominated by the air-breathing tetrapods^28^. Given the lack of high-resolution radioisotopic geochronology and biostratigraphy to “anchor” the ATS, we rely upon an assumption based on late Mesozoic and early Cenozoic carbon cycling studies, i.e. δ^13^C_carb_ minima correspond to eccentricity maxima^20^,^21^,^22^,^23^,^24^,^39^,^40^,^41^,^42^,^43^. Thus, the reversed eccentricity curve of La10d was tied at 247.95 Ma to a depth of 162.62 m (Fig. 2E,F). Using this approach, the S-S boundary^34^ in the Chaohu area, marked by an abrupt 4% positive shift in δ^13^C_carb_ at the top of fossil-rich black shale (pink line in Figs 1 and 2D–F), is constrained to 250.21 Ma, which compares well with the radioisotopic date of 250.55 ± 0.51 Ma^32^ for the earliest Spathian. The duration of the 1.36-m thick black shale (18.46 m to 19.82 m, see Supplementary Fig. S2A) below the S-S boundary was estimated to be 19.21 kyr using an astronomically calibrated average sedimentation rate of ~7.08 cm/kyr (see Supplementary Table S4). Although the exact stratigraphic level of the Spathian-Anisian boundary is unknown in the Chaohu area, it can be inferred at the Majiashan section to be at ~171.70 ± 3.72 m above the S-S boundary using an end-Spathian age of 247.32 ± 0.08 Ma^33^. In this case, the astronomically tuned time series constrains the duration of the Spathian interval to 2.89 ± 0.08 My, which falls within the uncertainty of the U-Pb estimated duration of 3.23 ± 0.60 My^32^,^33^,^44^.

According to our excavation at the Majiashan section, the lowest stratigraphic level of occurrence of marine reptiles in the Chaohu Fauna occurs in the Middle Member of the Nanlinghu Formation, ~117.00 m above the base of the Helongshan Formation (Fig. 1), and is constrained to ~248.81 Ma (Fig. 2). Abundant ichthyosauromorphs occur in the interval 135.83 m to 149.26 m above the base of the Helongshan Formation with a duration of ~279.79 kyr (Fig. 1), including *Chaohusaurus*, which was fully adapted to the marine aqueous habitat and yet maintained a birth posture typical of terrestrial reptiles, and *Cartorhynchus* with possible amphibious habits and suction-feeding behavior^45^. The most primitive Eosauropterygia^46^, which shows similarity to Middle Triassic Eosauropterygia, occurs ~156.12 m above the base of the Helongshan Formation (Fig. 1) and is constrained to ~248.10 Ma (Fig. 2). Ichthyosaurs were also found in the upper Spathian (Fig. 2).

## Discussion

Persistent eccentricity and obliquity cyclicity in the EHA of the tuned δ^13^C_carb_ data (see Supplementary Fig. S12) indicates astronomically-paced redistribution of carbon between surficial C reservoirs. Seven phases of δ^13^C_carb_ are identified from the minima of the filtered 405-kyr components (long-term eccentricity) (Fig. 2D). The good alignment between the amplitude envelope of the filtered 100-kyr components and the 405-kyr components in Phases 5 through 7 shows the robustness of the tuning results (Fig. 2E). The overall weaker short eccentricity and variable phase relationship observed in Phases 1 through 4 suggests a fundamental change in the response of seawater δ^13^C to astronomical forcing in the mid-Spathian, coincident with the onset of increased short-term δ^13^C_DIC_ volatility and a 2% decrease in δ^13^C_DIC_, the last of a stepped long-term decrease following the S-S boundary positive excursion. The amplification of obliquity (Fig. 2C) begins between the two abrupt δ^13^C shifts that define the longer-term decrease in the Spathian.

The response of the ocean carbon reservoir to astronomical forcing is largely dependent on the oceanic structure, which in turn responds to climate change^23^. Astronomically paced changes in ocean ventilation (oxygenation) lead to periodic releases of ^12^C-enriched carbon to the surface ocean. For greenhouse times, such as the Early Triassic^47^, the greater water column stratification of greenhouse oceans makes the ocean C budget more sensitive to orbital variations^20^,^23^,^24^. Together the shift in eccentricity phasing and obliquity amplification likely record a major restructuring of oceanic circulation (cf. refs 24 and 25) linked to climate amelioration during the Spathian^13^,^31^. A strongly stratified water column would have supported an expanded oxygen minimum zone and build up of a large dissolved organic carbon (DOC) reservoir in the deep ocean (cf., ref. 48). Turnover from such an oceanic state to a better and deeper ventilated ocean with major restructuring would have released large amounts of this ^12^C-enriched carbon pool to surficial C reservoirs and accelerated the rate of carbon cycling thus amplifying the response of seawater δ^13^C_DIC_ to eccentricity and obliquity forcing/pacing^20^,^21^. In this context, we interpret the overall higher δ^13^C_carb_ values (+2 to +4%) of the first 800 kyr (Phase 2–3) of the Spathian as recording strong water column stratification, high productivity, enhanced organic matter burial and likely build up of the deep ocean DOC reservoir promoted by the expanded oxygen minimum zone^13^. The subsequent change in the nature of eccentricity and obliquity cyclicity, along with the final abrupt shift to values that fluctuate around a stable mean (~−2%) characteristic of pre-perturbation thermocline δ^13^C_DIC_ values in the Majiashan slope environment^31^, constrains the timing of termination of O_2_-limited oceanic conditions to the mid-Spathian. The observation of obliquity amplification within phase 4, and continuing into phases 5 through 6, is consistent with the development of a high-latitude, oxygenated, intermediate to deep-water source perhaps in response to cooling and/or eustatic change (cf. ref. 49, 50, 51) that would have led to enhanced ventilation of the deep ocean. The near loss of long-term eccentricity δ^13^C_carb_ cyclicity in Phase 7, and weakening of obliquity, could capture the return to a fully stable well-oxygenated ocean sustained by vigorous circulation (cf. ref. 24).

The ATS indicates that the stratigraphically lowest marine reptile fossil in the Chaohu Fauna, and around the world^52^, occurs in the later portion of Phase 4 and has an age of ~248.81 Ma. This is the oldest known Mesozoic marine reptile found to date despite concerted but unsuccessful search efforts to find older fossils. Thus, the timing of reptilian invasion into the oceans was likely not much earlier than 248.81 Ma. This estimate constrains the post-extinction ecosystem recovery period to ~3.35 Myr after the PTB (at ~252.16 Ma, as recognized in ref. 32). The main reptile fossil beds that yield *Cartorhynchus*, a basal and potentially amphibious ichthyosauriform^45^, as well as abundant multi-clade marine reptile fossils, occur within Phases 5 and 6 (Fig. 2D–F). The Chaohu Fauna is the oldest marine fauna with tetrapod as top predators and a high marine ecosystem complexity, as far as we know.

Previous studies of Early Triassic benthic faunas and vertebrate fossil localities with limited marine ecosystem complexity^53^,^54^ supported the hypothesis of a delayed biotic recovery^2^,^4^. The appearance of marine reptiles^45^,^46^ and the Triassic middle fish fauna (TMFF)^55^ in Chaohu in the middle Spathian marks the initiation of the establishment of a new Meso-Cenozoic marine ecosystem and thus indicates a high degree of biotic recovery following the end-Permian Mass Extinction at this time^52^. The temporal relationship between the occurrence of the oldest marine reptile fossil and obliquity amplification (Phase 4) and the appearance of multi-clade marine reptiles during the subsequent change in the nature of the eccentricity modulation (Phases 5–6), temporally linked to the final phase of a two-step decrease in seawater δ^13^C_DIC_, indicates a mechanistic link between the appearance and diversification of the marine reptiles and the major change in ocean structure and carbon cycling. We attribute the biotic recovery and initiation of a new marine ecosystem to the final breakdown of ocean stratification and the onset of a high-latitude deep-water source, which would have enhanced ventilation of the deep ocean. Together these changes in ocean structure would have stimulated surface ocean primary productivity promoting the diversification of the marine reptiles. They further suggest that reptiles may have first invaded Panthalassa in the middle Spathian in response to this reorganization.

Marine reptiles introduced a new pattern of shallow marine nutrient circulation in the Early Triassic that may have played a critical role in the post-extinction build-up of the marine ecosystem, by feeding at various depths and defecating near the sea surface^52^. The post-extinction recovery of the forest ecosystem in the middle Spathian is contemporaneous with that of the marine ecosystem^56^,^57^ as would be anticipated if changes in ocean circulation were driven by climate amelioration. Ultimately, stabilization of oceanic conditions in the Early Triassic, which involved a change in the response of carbon cycling to astronomical forcing, likely accelerated the building of the new Meso-Cenozoic marine ecosystem.

In summary, the first occurrence of the Mesozoic marine reptiles is approximately coincident with a shift in astronomically-paced marine carbon cycling, and a ~2% shift in seawater DIC, which indicates that the South China seas remained inhabitable to large air-breathing marine predators following the end-Permian Mass Extinction until a major rearrangement of oceanic circulation. This change led to intensified ventilation and restored well-oxygenated conditions, which is mechanistically linked to astronomically driven changes in C cycling between Earth’s surface reservoirs. The distribution of diversified marine reptiles along the coasts of the Panthalassan Sea in the late Spathian^54^ was likely fueled by the climate amelioration, changing ocean structure, and increased surface ocean primary productivity in the middle Spathian. The same factors may have encouraged reptiles to invade the sea. Ultimately, stabilization of oceanic conditions in the Early Triassic may have involved climate-life interactions in which a change in the astronomical pacing of climate and carbon cycling among the Earth’s surface carbon reservoirs accelerated the building of the new Meso-Cenozoic marine ecosystem.

## Methods

Carbonate samples were collected and analyzed at a spacing of ~5–10 kyr from the Majiashan section, Chaohu spanning the late Smithian to Spathian in the Isotope Lab of Nanjing Institute of Geology and Paleontology, Chinese Academy of Sciences with an analytical precision of <0.03%. δ^18^O_carb_, δ^13^C_carb_ δ^13^C_org_ with an analytical precision of <0.1% and wt.% CaCO_3_ of microdrilled samples were generated in the Stan Margolis Stable Isotope Lab, UC Davis. The δ^13^C_carb_ data were prepared and analyzed in the R software package “Astrochon” (Meyers, 2014; R Core Team, 2015).

## Additional Information

**How to cite this article**: Fu, W. *et al*. Eccentricity and obliquity paced carbon cycling in the Early Triassic and implications for post-extinction ecosystem recovery. *Sci. Rep*. **6**, 27793; doi: 10.1038/srep27793 (2016).

## Supplementary Material

Supplementary Information

## References

[b1] Pruss S., Fraiser M. & Bottjer D. J. Proliferation of Early Triassic wrinkle structures: Implications for environmental stress following the end-Permian mass extinction. Geology 32, 461–464 (2004).

[b2] Payne J. L. . Large perturbations of the carbon cycle during recovery from the End-Permian Extinction. Science 305, 506–509 (2004). 15273391 10.1126/science.1097023

[b3] Stanley S. M. Evidence from ammonoids and conodonts for multiple Early Triassic mass extinctions. Proceedings of the National Academy of Sciences 106, 15264–15267 (2009). 10.1073/pnas.0907992106 PMC2741239 19721005

[b4] Chen Z.-Q. & Benton M. J. The timing and pattern of biotic recovery following the end-Permian mass extinction. Nature Geosci 5, 375–383 (2012).

[b5] Knoll A. H., Bambach R. K., Payne J. L., Pruss S. & Fischer W. W. Paleophysiology and end-Permian mass extinction. Earth and Planetary Science Letters 256, 295–313 (2007).

[b6] Sun Y. . Lethally Hot Temperatures During the Early Triassic Greenhouse. Science 338, 366–370 (2012). 23087244 10.1126/science.1224126

[b7] Romano C. . Climatic and biotic upheavals following the end-Permian mass extinction. Nature Geosci 6, 57–60 (2013).

[b8] Wignall P. B. & Hallam A. Griesbachian (Earliest Triassic) palaeoenvironmental changes in the Salt Range, Pakistan and southeast China and their bearing on the Permo-Triassic mass extinction. Palaeogeography, Palaeoclimatology, Palaeoecology 102, 215–237 (1993).

[b9] Wignall P. B. . An 80 million year oceanic redox history from Permian to Jurassic pelagic sediments of the Mino-Tamba terrane, SW Japan, and the origin of four mass extinctions. Global and Planetary Change 71, 109–123 (2010).

[b10] Grice K. . Photic Zone Euxinia During the Permian-Triassic Superanoxic Event. Science 307, 706–709 (2005). 15661975 10.1126/science.1104323

[b11] Algeo T. J. . Changes in productivity and redox conditions in the Panthalassic Ocean during the latest Permian. Geology 38, 187–190 (2010).

[b12] Luo G. . Isotopic evidence for an anomalously low oceanic sulfate concentration following end-Permian mass extinction. Earth and Planetary Science Letters 300, 101–111 (2010).

[b13] Song H. . Early Triassic seawater sulfate drawdown. Geochimica et Cosmochimica Acta 128, 95–113 (2014).

[b14] Suzuki N., Ishida K., Shinomiya Y. & Ishiga H. High productivity in the earliest Triassic ocean: black shales, Southwest Japan. Palaeogeography, Palaeoclimatology, Palaeoecology 141, 53–65 (1998).

[b15] Meyer K. M., Yu M., Jost A. B., Kelley B. M. & Payne J. L. δ13C evidence that high primary productivity delayed recovery from end-Permian mass extinction. Earth and Planetary Science Letters 302, 378–384 (2011).

[b16] Twitchett R. J., Krystyn L., Baud A., Wheeley J. R. & Richoz S. Rapid marine recovery after the end-Permian mass extinction event in the absence of marine anoxia. Geology 32, 805–808 (2004).

[b17] Song H., Tong J. & Chen Z. Q. Evolutionary dynamics of the Permian–Triassic foraminifer size: Evidence for Lilliput effect in the end-Permian mass extinction and its aftermath. Palaeogeography, Palaeoclimatology, Palaeoecology 308, 98–110 (2011).

[b18] Rieppel O. Sauropterygia I. In Handbook of Paleoherpetology Vol. 12 (Verlag Dr. Friedrich Pfeil, 2000).

[b19] McGowan C. & Motani R. Ichthyopterygia In Handbook of Paleoherpetology Vol. 8 (Verlag Dr. Friedrich Pfeil, 2003).

[b20] Zachos J. C. . Climate response to orbital forcing across the Oligocene-Miocene Boundary. Science 292, 274–278 (2001). 11303100 10.1126/science.1058288

[b21] Cramer B. S., Wright J. D., Kent D. V. & M. P. Orbital climate forcing of δ^13^C excursions in the late Paleocene–early Eocene (chrons C24n–C25n). Paleoceanography 18, 1097 (2003).

[b22] Pälike H. . The heartbeat of the Oligocene climate system. Science 314, 1894–1898 (2006). 17185595 10.1126/science.1133822

[b23] Wang P., Tian J. & Lourens L. J. Obscuring of long eccentricity cyclicity in Pleistocene oceanic carbon isotope records. Earth and Planetary Science Letters 290, 319–330 (2010).

[b24] Giorgioni M. . Orbital control on carbon cycle and oceanography in the mid-Cretaceous greenhouse. Paleoceanography 27, PA1204 (2012).

[b25] Huang C., Tong J., Hinnov L. & Chen Z. Q. Did the great dying of life take 700 k.y.? Evidence from global astronomical correlation of the Permian-Triassic boundary interval. Geology 39, 779–782 (2011).

[b26] Wu H. . Time-calibrated Milankovitch cycles for the late Permian. Nature Communication 4: 2453 (2013). 10.1038/ncomms3452 PMC3778519 24030138

[b27] Li M. . Astronomical tuning of the end-Permian extinction and the Early Triassic Epoch of South China and Germany. Earth and Planetary Science Letters 441, 10–25 (2016).

[b28] Erwin D. H. The Permo-Triassic extinction. Nature 367, 231–236 (1994).

[b29] Kump L. R. & Arthur M. A. Interpreting carbon-isotope excursions: carbonates and organic matter. Chemical Geology 161, 181–198 (1999).

[b30] Sepkoski J. J., Bambach R. K., Raup D. M. & Valentine J. W. Phanerozoic marine diversity and the fossil record. Nature 293, 435–437 (1981).

[b31] Song H. . Large vertical δ^13^C_DIC_ gradients in Early Triassic seas of the South China craton: Implications for oceanographic changes related to Siberian Traps volcanism. Global and Planetary Change 105, 7–20 (2013).

[b32] Ovtcharova M. . New Early to Middle Triassic U–Pb ages from South China: Calibration with ammonoid biochronozones and implications for the timing of the Triassic biotic recovery. Earth and Planetary Science Letters 243, 463–475 (2006).

[b33] Lehrmann D. J. . Timing of recovery from the end-Permian extinction: Geochronologic and biostratigraphic constraints from south China. Geology 34, 1053–1056 (2006).

[b34] Tong J. . Events during Early Triassic recovery from the end-Permian extinction. Global and Planetary Change 55, 1–3 (2007).

[b35] Galfetti T. . Late Early Triassic climate change: Insights from carbonate carbon isotopes, sedimentary evolution and ammonoid paleobiogeography. Palaeogeography, Palaeoclimatology, Palaeoecology 243, 394–411 (2007).

[b36] Meyers S. R. & Sageman B. B. Quantification of deep-time orbital forcing by average spectral misfit. American Journal of Science 307, 773–792 (2007).

[b37] Laskar J. . A long-term numerical solution for the insolation quantities of the Earth. Astronomy and Astrophysics 428, 261–285 (2004).

[b38] Laskar J., Fienga A., Gastineau M. & Manche H. La2010: A new orbital solution for the long-term motion of the Earth. Astronomy and Astrophysics 532, A89 (2011).

[b39] Woodruff F. & Savin S. Mid-Miocene isotope stratigraphy in the deep sea: High-resolution correlations, paleoclimatic cycles, and sediment preservation. Paleoceanography 6, 755–806 (1991).

[b40] Flower B. P. & Kennett J. P. Middle Miocene deepwater paleoceanography in the southwest Pacific: Relations with East Antarctic Ice Sheet development. Paleoceanography 10, 1095–1112 (1995).

[b41] Billups K., Pälike H., Channell J. E. T., Zachos J. C. & Shackleton N. J. Astronomic calibration of the late Oligocene through early Miocene geomagnetic polarity time scale. Earth and Planetary Science Letters 224, 33–44 (2004).

[b42] Holbourn A., Kuhnt W., Schulz M. & Erlenkeuser H. Impacts of orbital forcing and atmospheric carbon dioxide on Miocene ice-sheet expansion. Nature 438, 483–487 (2005). 16306989 10.1038/nature04123

[b43] Holbourn A., Kuhnt W., Schulz M., Flores J.-A. & Andersen N. Orbitally-paced climate evolution during the middle Miocene “Monterey” carbon-isotope excursion. Earth and Planetary Science Letters 261, 534–550 (2007).

[b44] Galfetti T. . Timing of the Early Triassic carbon cycle perturbations inferred from new U–Pb ages and ammonoid biochronozones. Earth and Planetary Science Letters 258, 593–604 (2007).

[b45] Motani R. . A basal ichthyosauriform with a short snout from the Lower Triassic of China. Nature 517, 485–488 (2015). 25383536 10.1038/nature13866

[b46] Jiang D. Y. . The Early Triassic eosauropterygian Majiashanosaurus discocoracoidis, gen. et sp. nov. (Reptilia, Sauropterygia), from Chaohu, Anhui Province, People’s Republic of China. Journal of Vertebrate Paleontology 34, 1044–1052 (2014).

[b47] Preto N., Kustatscher E. & Wignall P. B. Triassic climates — State of the art and perspectives. Palaeogeography, Palaeoclimatology, Palaeoecology 290, 1–10 (2010).

[b48] Sexton P. F. . Eocene global warming events driven by ventilation of oceanic dissolved organic carbon. Nature 471, 349–352 (2011). 21412336 10.1038/nature09826

[b49] Bornemann A. . Isotopic evidence for glaciation during the Cretaceous super greenhouse. Science 319, 189–192 (2008). 18187651 10.1126/science.1148777

[b50] Westerhold T. & Röhl U. High resolution cyclostratigraphy of the early Eocene-New insights into the origin of the Cenozoic cooling trend. Clim. Past 5, 309–327 (2009).

[b51] Meyers S. R., Sageman B. B. & Arthur M. A. Obliquity forcing of organic matter accumulation during Oceanic Anoxic Event 2. Paleoceanography 27, 3212 (2012).

[b52] Motani R. . Lunge feeding in early marine reptiles and fast evolution of marine tetrapod feeding guilds. Scientific Reports 5, 8900 (2015). 25754468 10.1038/srep08900 PMC4354009

[b53] Foster W. J. & Twitchett R. J. Functional diversity of marine ecosystems after theLate Permian mass extinction event. Nature Geosci 7, 233–238 (2014).

[b54] Scheyer T. M., Romano C., Jenks J. & Bucher H. Early Triassic Marine Biotic Recovery: The Predators’ Perspective. PLoS ONE 9, 1–20 (2014). 10.1371/journal.pone.0088987 PMC3960099 24647136

[b55] Tintori A., Hitij T., Jiang D., Lombardo C. & Sun Z. Triassic actinopterygian fishes: the recovery after the end-Permian crisis. Integrative Zoology 9, 394–411 (2014). 24148549 10.1111/1749-4877.12077

[b56] Looy C. V., Brugman W. A., Dilcher D. L. & Visscher H. The delayed resurgence of equatorial forests after the Permian-Triassic ecologic crisis. Proceedings of the National Academy of Sciences of the United States of America 96, 13857–13862 (1999). 10570163 10.1073/pnas.96.24.13857 PMC24155

[b57] Saito R. . A terrestrial vegetation turnover in the middle of the Early Triassic. Global and Planetary Change 105, 152–159 (2013).

