# Supplementary material for: Eccentricity and obliquity paced carbon cycling in the Early Triassic and implications for post-extinction ecosystem recovery
Source: Sci Rep. 2016 Jun 13;6:27793. doi: 10.1038/srep27793 (PMC4904238; doi:10.1038/srep27793)
Supplement: Supplementary Information [file srep27793-s1.pdf]

Supporting Material for

Eccentricity and obliquity paced carbon cycling in the Early Triassic and implications for post-extinction ecosystem recovery

Wanlu Fu[1], Da-yong Jiang[*,1], Isabel P. Montañez[*,2], Stephen R. Meyers[3], Ryosuke Motani[2], and Andrea Tintori[4]

[1]Laboratory of Orogenic Belt and Crustal Evolution, Ministry of Education; Department of Geology and Geological Museum, Peking University, Yiheyuan Street. 5, Beijing 100871, P.R. China, wanlufu@pku.edu.cn, djiang@pku.edu.cn;

[2]Department of Earth and Planetary Sciences, University of California, Davis, One Shields Avenue, Davis, California 95616, U.S.A., ipmontanez@ucdavis.edu, rmotani@ucdavis.edu;

[3]Department of Geoscience, University of Wisconsin, 1215 West Dayton St, Madison, WI 53076, smeyers@geology.wisc.edu

[4]Dipartimento di Scienze della Terra, Università degli Studi di Milano, Via Mangiagalli 34-20133 Milano, Italy, andrea.tintori@unimi.it

This file includes:

Material and method

Figures S1-S12

Table S1-S5

References

R_analysis

Materials and methods

Majiashan section. The stratigraphic succession in Chaohu, Anhui Provice, south China (31.62° N, 117.82° E), is one of the most significant Early Triassic marine sections globally. With a complete Olenekian succession, it yields the best-preserved, abundant and diverse Early Triassic marine reptile fossils[1-2]. The Majiashan section was deposited in a deep-water (200 to 500 m) slope setting on the northern border of the Yangtze Platform where the eastern Tethyan and western Panthalassa were connected[3-4]. Cyclostratigraphic studies of the Majiashan section identified meter-scale stratigraphic packages interpreted to be shallowing-upward cycles (Fig. S1)[5].

The section can be subdivided into 4 lithofacies. (1) 0-20 m: Highly cyclic sedimentation of alternating pyrite-rich marl and bivalve wackestone of the Helongshan Formation spanning the Smithian-Spathian boundary (SSB), which is characterized by a 1.36-m thick organic-rich fossil-bearing shale (Fig. S2A). Fossils across the SSB are well preserved together with pyrite in black shales and in calcareous concretions, including fishes, bivalves, gastropods and ammonites (Fig. S2B). The presence of a rich vertebrate bearing bed with no reptile remains at all provides a potential time constrain to the marine reptile origin. (2) 20-82 m: Massive limestone interbedded with nodular limestones of the Lower Nanlinghu Formation, recording a relative sea level fall. The carbonate-rich laminae of the nodular limestone in this lithofacies exhibit clasts of microbial peloidal bindstone, likely sourced from the shallower water carbonate platform (Fig. S2C-D, Fig. S3). (3) 82-128 m: Massive ammonite-bearing nodular limestones alternate with thin-bedded micritic limestones with rare bioclasts (Fig. S2E-F). The nodular limestones are similar to those of the Lower Nanlinghu Formation and terminate in this sequence. (4) 128-203 m: Highly cyclic deposits of marl alternating with carbonate mudstones (micrite) and overlying shales interbedded with laminated mudstones, recording a relative sea-level rise (Fig. S1B; Fig. S2G-H). Abundant reptile remains have been found in lithofacies 4. In this case, lithofacies 1 and 4 yield vertebrates but none remains were discovered in lithofacies 2 and 3.

The global Smithian-Spathian boundary has not been yet resolved anywhere in the world. In the Chaohu area, South China, the boundary is identified by the first

occurrence of conodont *Neospathodus pingdingshanensis* in the Pingdingshan succession, which has not been correlated globally[6]. Either has ammonoid zonation been widely correlated[7]. A major positive excursion in $\delta^{13}C_{carb}$ corresponds to a lithofacies boundary at the SSB and provides a significant global correlation tool[8-9].

Stable isotope analysis. Limestone, mudstone and calcareous shale were sampled at a 10-cm stratigraphic resolution from the uppermost Smithian through upper Spathian succession at Majiashan. Sampling resolution at the Smithian-Spathian transition was 1-cm spacing. To rule out diagenetic alteration, samples were trimmed to remove veins, fractures, and the weathered surface and ground to 200-mesh size in ball mill. For limestone beds greater than 1 m-thick, rock powder of five consecutive samples was homogenized to provide 50 cm spacing. Stable isotope analysis of whole-rock samples were analyzed at 24°C ± 1°C using a MAT 253™ stable isotope ratio mass spectrometer housed in the Isotope Lab of Nanjing Institute of Geology and Paleontology, Chinese Academy of Sciences with an analytical precision of <0.03‰. In order to evaluate for potential diagenetic alteration of the depositional $\delta^{13}C$ values of bulk-rock samples, we analyzed, for a select subset of the samples used for whole-rock analysis, the $\delta^{13}C_{carb}$ and wt.% carbonate of microdrilled samples, which were petrographically identified as 'best preserved' micrite, (Fig. S4). Micro-drilled samples (30 to 60 μg) were roasted at 375 °F *in vacuo* to remove organic volatiles. $\delta^{18}O_{carb}$ and $\delta^{13}C_{carb}$ values were determined using a Fisons Optima IRMS with a 90°C Isocarb common acid bath autocarbonate system in the Stan Margolis Stable Isotope Lab, UC Davis. Analytical precision for both $\delta^{18}O$ and $\delta^{13}C$ is <±0.1‰ (1σ). All stable isotope values are reported relative to Pee Dee Belemnite (PDB) using standard delta notation (Table S1). The overlap of bulk rock and micro-drilled powder values (Fig. S4A), the lack of correlation between $\delta^{18}O_{carb}$, $\delta^{13}C_{carb}$ (Fig. S4B), wt.% carbonate, and $\delta^{13}C_{carb}$ (Fig. S4 C) and the coupling of $\delta^{13}C_{carb}$ and $\delta^{13}C_{org}$ (Fig. S5) indicate minimal diagenetic alteration of the observed values and provide confidence that the whole-rock analyses provide a robust record of the seawater $\delta^{13}C_{carb}$ (cf. Ref. 10, 11).

Three major $\delta^{13}C_{carb}$ shifts, which are synchronous with major lithologic

boundaries, define four phases of the $\delta^{13}C_{carb}$ record with average $\delta^{13}C_{carb}$ values of -5 ‰, +2‰, 0 ‰, and -2‰ (Fig. 1). These shifts are potentially linked to changes in water column stratification and oceanic circulation conditions. Major long-term $\delta^{13}C_{carb}$ minima delineate 7 large-scale oscillations in the $\delta^{13}C_{carb}$ record (Fig. S6). Global comparison of time-equivalent $\delta^{13}C_{carb}$ records reveals similar long-term features[8, 9, 12].

Astrochronologic analysis. To further constrain the observed $\delta^{13}C_{carb}$ variations and the chronology of the SSB and the marine reptile beds, we performed astrochronologic testing on the high-resolution $\delta^{13}C_{carb}$ data. The $\delta^{13}C_{carb}$ data were prepared and analyzed using the R software package "Astrochron" [13].

(1) Radioisotopic constraints. Available geochronology was used to calculate the approximate duration of the interval and to assess the potential time scale of the observed oscillations in the $\delta^{13}C_{carb}$ data. A U/Pb age of 250.55 ± 0.51 Ma (2σ analytical+tracer uncertainty) for the earliest Spathian within the ammonoid *Tirolites*/*Columbites* Zone[14] from northwestern Guangxi, South China is correlated to the Lower Nanlinghu Formation in the Majiashan section by ammonoid assemblage and a positive $\delta^{13}C$ excursion (Fig. 1). The U/Pb ages of 247.38 ± 0.10 Ma (1σ analytical uncertainty) and 247.32 ± 0.08 Ma (1σ analytical uncertainty) for the latest Spathian within the conodont assemblage of *N. homeri* and *N. abruptus* from the Great Bank of Guizhou, China[15] cannot be unambiguously correlated to the uppermost Nanlinghu Formation due to the absence of a positive $\delta^{13}C$ excursion and the low resolution of conodont assemblages[6]. However, an estimated duration of < 3.23 ± 0.60 My for the Nanlinghu Formation can be calculated (see below), thus indicating that the time scale for the observed very strong long-term oscillations in the $\delta^{13}C_{carb}$ data (Fig. 1) is 10^5 years.

(2) Data preparation. The long-term trend in the $\delta^{13}C_{carb}$ data is removed with a LOWESS smoother of 0.08, and subsequently 10 extremely negative "outliers" were removed using a threshold of -2‰. The mean sampling interval of the subsequent data set is 33 cm and the median sampling interval is 19 cm. In order to obtain evenly spaced $\delta^{13}C_{carb}$ data prior to time series analysis, the detrended data were linearly interpolated to a constant 15 cm resolution. This fine sampling grid is appropriate for the MTM Harmonic F-test that is

employed to evaluate peak significance (in contrast to red noise tests, which are sensitive to over-interpolation) (Fig. S7A-B).

(3) Time-frequency analysis. Evolutive Harmonic Analysis (EHA; Fig. S7C-D) of the prepared $\delta^{13}C_{carb}$ data provides an evaluation of changes in the spectral features through depth/time. EHA employs five 3π DPSS tapers, and a moving window of 12 m. Each window is linearly detrended and the result is evaluated up to the mean Nyquist frequency of 1.504221 cycle/m. The EHA method provides estimates of the amplitudes of periodic terms and includes a significance test for phase coherent sinusoids (the MTM Harmonic F-test) [16].

(4) Astrochronologic testing. The Evolutive Average Spectral Misfit method[17-19] (E-ASM; five 3π tapers; searching to the mean Nyquist frequency of 1.504221 cycles/m) was used to test a range of plausible timescales and simultaneously evaluate the reliability of the presence of astronomical cycles. The precession and obliquity target periods were determined from La04[20] using the interval from 248.00-249.00 Ma: 40.49 kyr (O1), 32.79 kyr (O2), 20.70 kyr (P1), 19.69 kyr (P2) and 17.06 kyr (P3). The eccentricity target periods were determined from La10d[21] using a slightly larger interval of 247.00-249.00 Ma, for better estimates of these longer periods: 400.00 kyr (E1), 131.58 kyr (E2), 99.01 kyr (E3).

The $\delta^{13}C_{carb}$ MTM Harmonic F-test results of the EHA in Figure S7D are evaluated using a grid of 200 sedimentation rates spanning 3 cm/kyr to 12 cm/kyr (log scaling of sedimentation rate grid). This range of sedimentation rates encompasses the long-term average sedimentation rate for the section based on the available geochronology (see section 1, Radioisotopic constraints). Duration of 3.23 ± 0.53 Ma (2σ analytical uncertainty) is estimated to correspond to a thickness of 180-190 m, and considering the stated uncertainties, this indicates a long-term average sedimentation rate of 4.79 cm/kyr to 7.04 cm/kyr.

All spectral peaks that met the 90% F-test confidence level (Fig. S7D) were evaluated using E-ASM, and Monte Carlo significance testing utilizing 100,000 simulations. Results with Null Hypothesis Significance Levels (Ho-SL) less than or equal to 0.1% were identified; note that this threshold is more conservative than the critical significance level of 0.5%[16]. Detailed results are shown in Table S3 and Fig. 2B.

(5) Astronomical tuning. Frequency domain minimal tuning[22] was applied to generate a floating astronomical time scale. Guided by the E-ASM results for specific intervals (Fig. S8), the average value of the detected short eccentricity terms (E2/2+E3/2=115.30 kyr) was tracked; the average short-term eccentricity was selected for minimal tuning because it is continuous and strongly expressed in the time series (Fig. S9, Table S3). The sedimentation rate curve derived from the spatial frequency modulation of the identified short-eccentricity term was integrated to obtain a depth-time map (Fig. S10, Table S4), and the tuned series was interpolated using a sampling grid of 7 kyr (the median sampling interval is 3.5 kyr and mean sampling interval is 5.8 kyr). Multi-taper method spectral analysis (MTM) and EHA results of the 115.30-kyr-tuned data (Fig. 2C-D, Fig. S11, Fig. S12, Table S5) provide further evidence for the presence of precession, obliquity and eccentricity cycles in the study interval, supporting the minimal tuning.

Periods of peaks that achieve the 90% AR1 CL are 500.06 kyr, 432.49 kyr, 242.45 kyr, 105.97 kyr, 61.31 kyr, 40.82 kyr, 36.04 kyr, 25.60 kyr, 18.56 kyr, 14.51 kyr (Fig. 2B).

(6) Bandpass filtering and amplitude modulation assessment. The long-term eccentricity and short-term eccentricity cycles (Table S5) were extracted from the tuned $\delta^{13}C_{carb}$ data using bandpass filters of 0.0015 to 0.0028 cycles/kyr and 0.006 to 0.011 cycles/kyr, respectively. The instantaneous amplitude envelope (Fig. 2E in dotted line) of the $\delta^{13}C_{carb}$ short eccentricity signal was determined via Hilbert Transform of the stratigraphic series to compare with the observed $\delta^{13}C_{carb}$ long eccentricity signal.

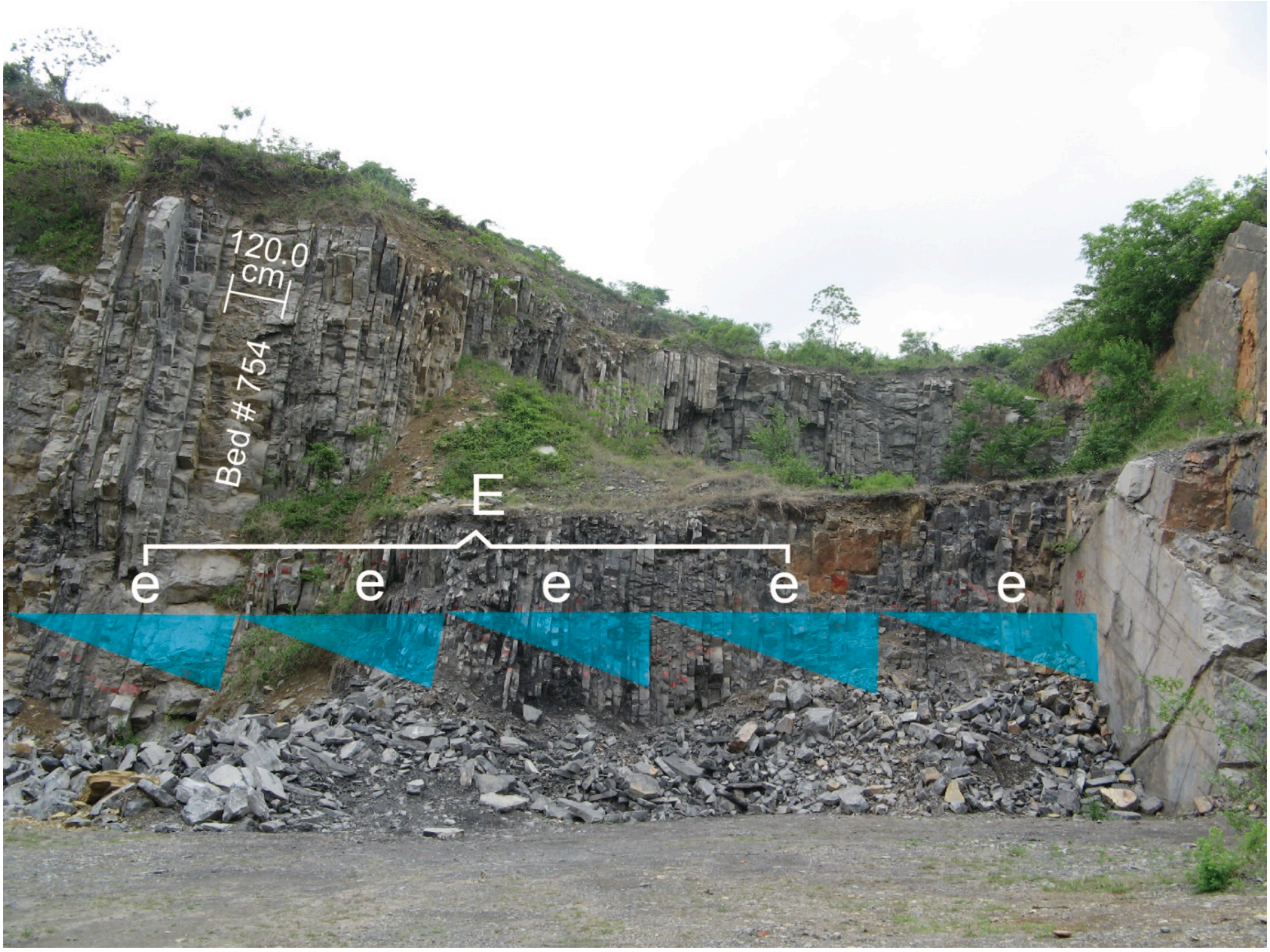

Figure S1. Stratigraphic cyclicity in the Majiashan succession. Up to the right.

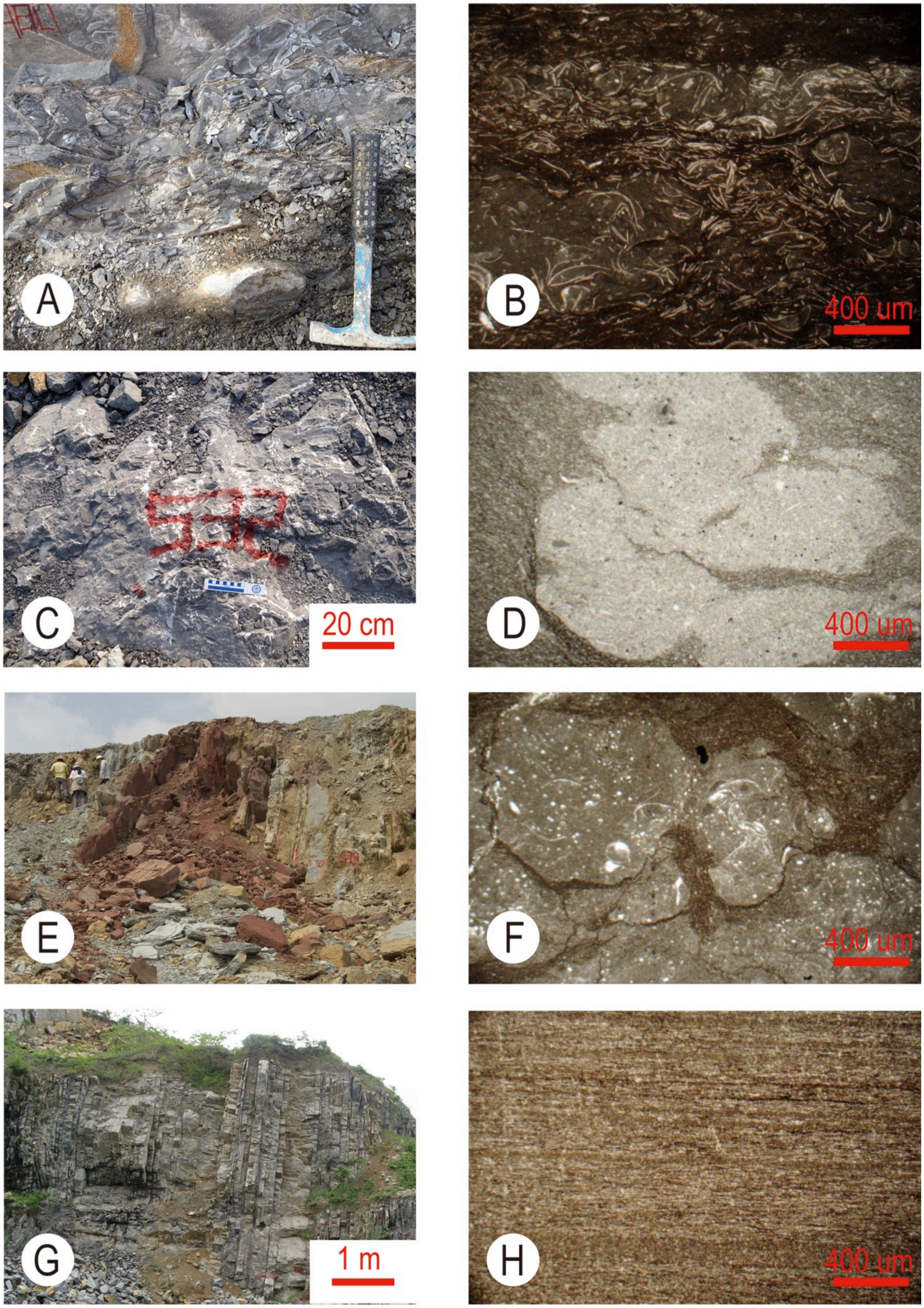
A
B
400 um
C
20 cm
D
400 um
E
F
400 um
G
1 m
H
400 um

Figure S2. Lithofacies and microfacies of Majiashan succession. Stratigraphic up is from top to bottom in A, from bottom to top B, D, F, H and from left to right in C, E and G. A-B. Fossil-bearing shale at the Smithian-Spathian boundary. C. Massive limestone of the Lower Nanlinghu Formation. D. Photomicrograph of nodular limestone of the Lower Nanlinghu Formation. E-F. Massive ammonite-bearing nodular limestone of the Middle Nanlinghu Formation (82-128 m). G. Cyclic deposits of marl alternating with micritic mudstones of the Upper Nanlinghu Formation. H. Photomicrograph of laminated mudstones of the Upper Nanlinghu Formation.

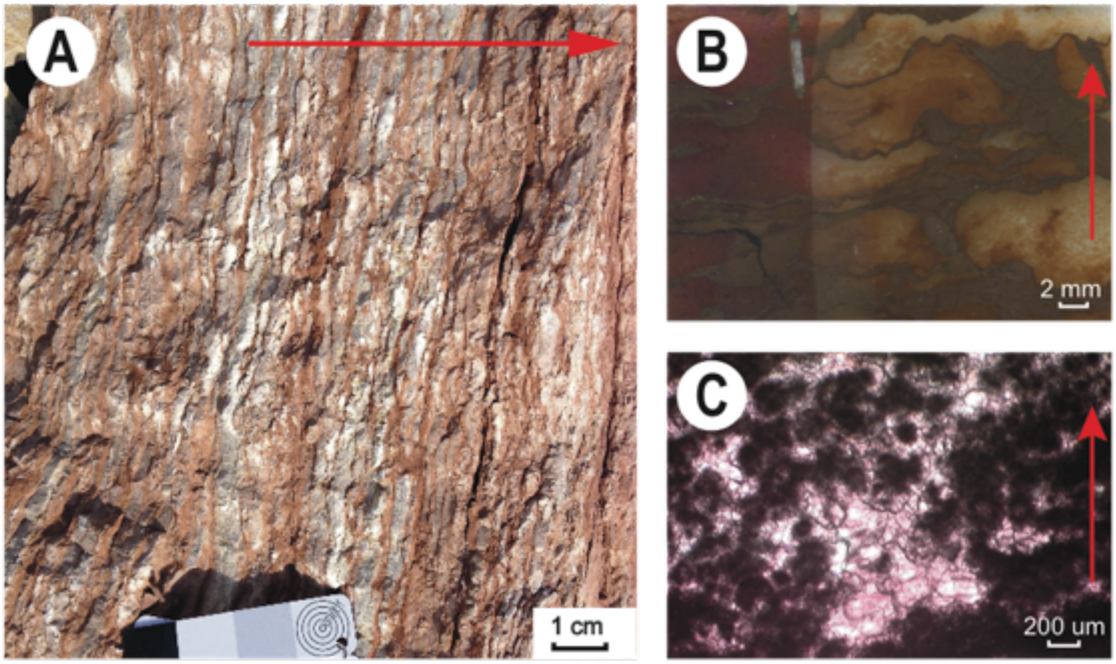

Figure S3. Nodular limestone of Lower Nanlinghu Formation. A. Nodular limestone composed of alternating red mud-rich and grey carbonate-rich laminae. B. Photomicrograph of pull-apart structures in nodular limestones that developed by gravity-driven sliding of coherent but unlithified sediment, indicative of a slope environment. Discoloration on the left is Alizarin Red staining of the carbonate. C. Photomicrograph exhibiting microbial peloidal bindstone composition of the carbonate-rich laminae. Red arrows in A, B and C indicate stratigraphic up.

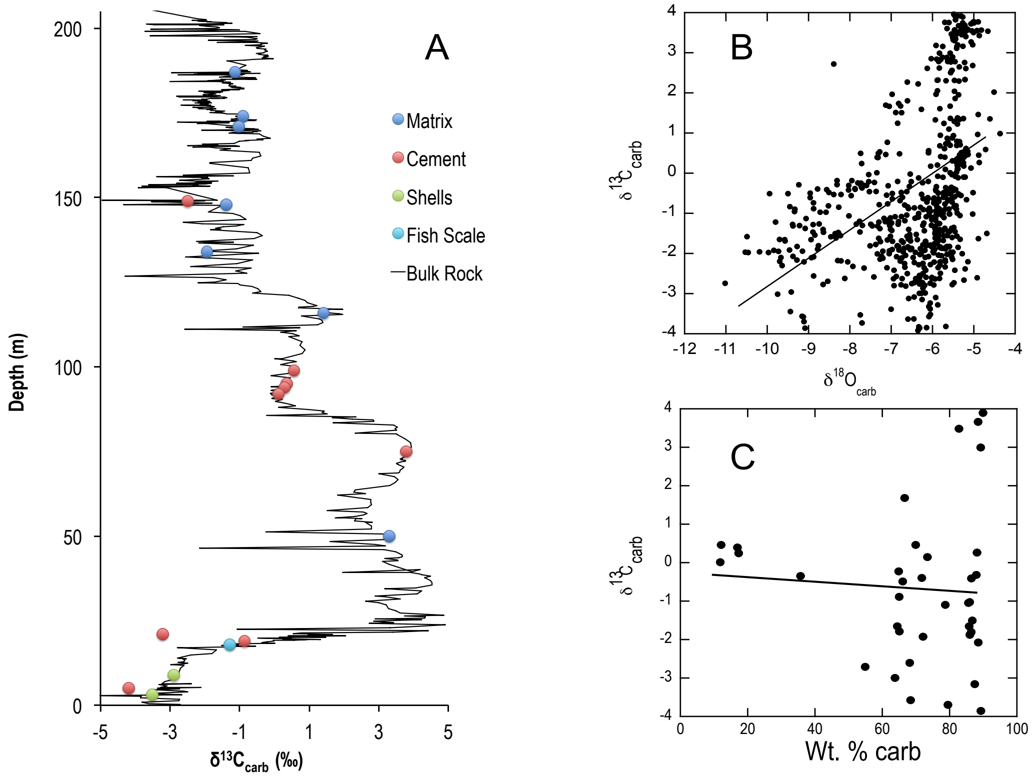

Figure S4. Diagenetic analysis of the Majiashan $\delta^{13}C_{carb}$ data. (A) Microdrilled sample $\delta^{13}C_{carb}$ values (colored symbols) superimposed on the whole rock $\delta^{13}C_{carb}$ trendline. (B) Crossplot of $\delta^{18}O_{carb}$ versus $\delta^{13}C_{carb}$ (r^2=0.18). (C) Crossplot of Wt. % carbonate versus $\delta^{13}C_{carb}$ ($r^2 < 0.01$).

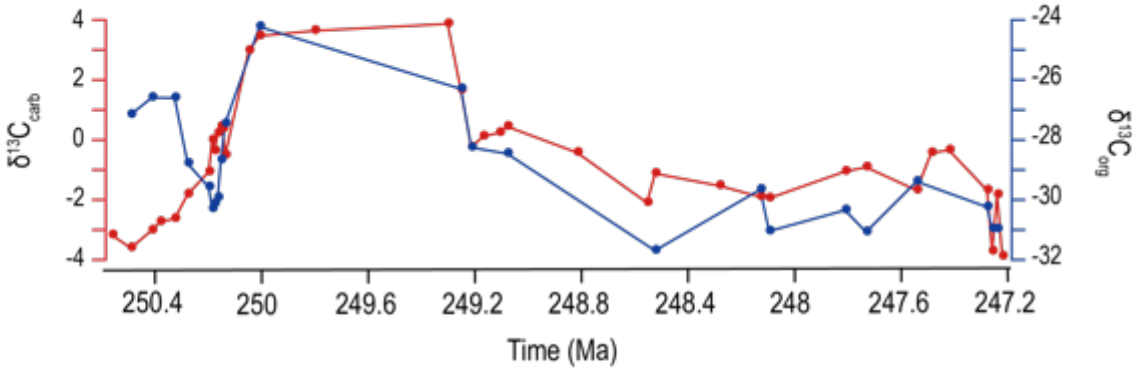

Figure S5. Temporal distribution of $\delta^{13}C_{carb}$ (red) and $\delta^{13}C_{org}$ (blue). Note that the timeline was based on astronomical tuning.

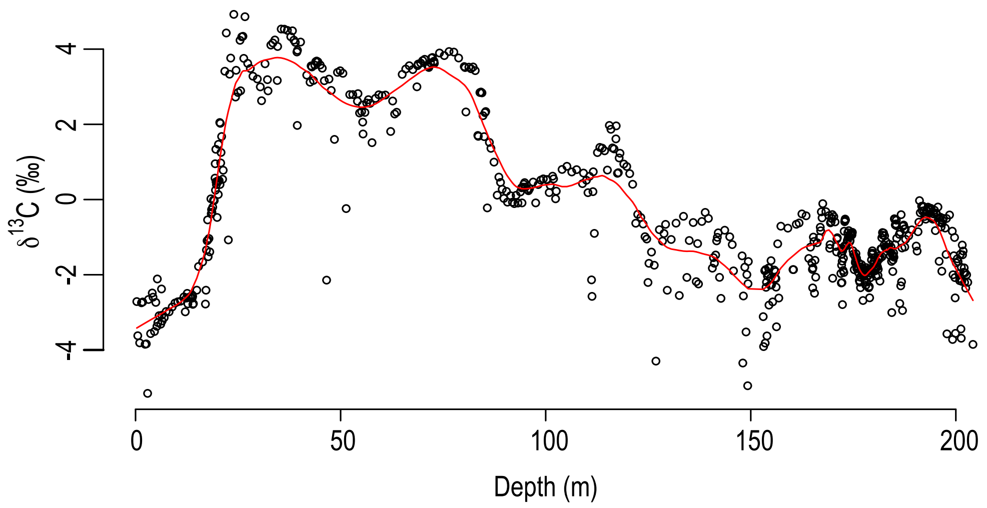

Figure S6. Majiashan section $\delta^{13}C_{carb}$ data with long-term trend determined using a LOWESS fit (red line), using a smoothing parameter of 0.08 (8% of the series length).

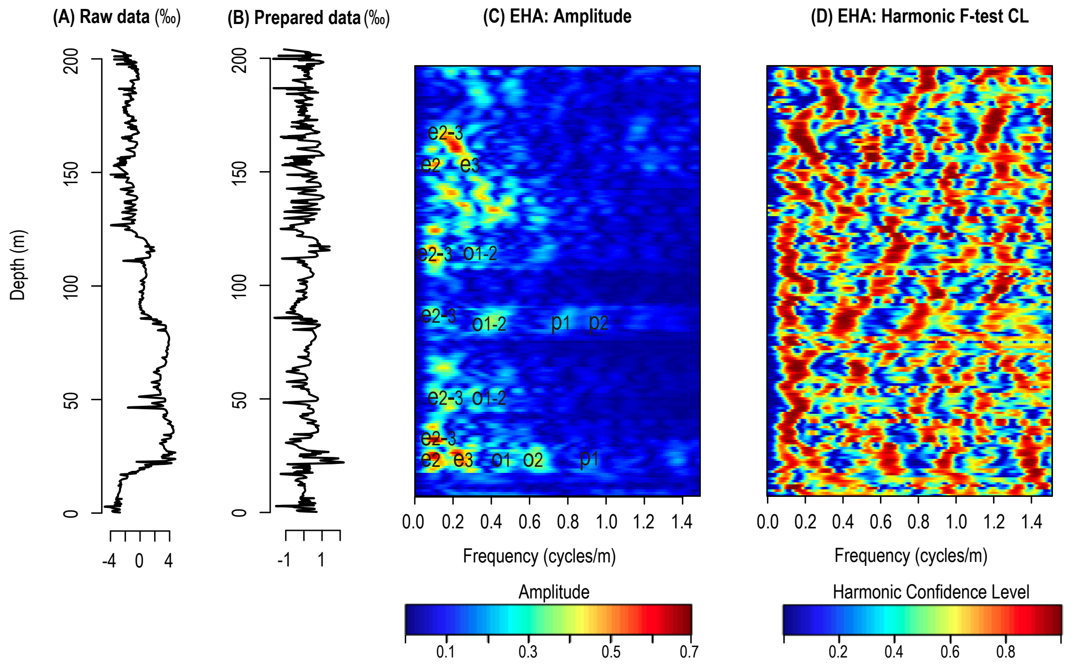

Figure S7. Evolutive harmonic analysis (EHA) of the Majiashan $\delta^{13}C_{carb}$ data. The detrended carbon isotopic data is linearly interpolated to a constant sample spacing of 0.15 m prior to analysis, following the removal of 10 outliers. EHA employs five 3π DPSS tapers and a moving window of 12 m. Each window is linearly detrended and the result is evaluated up to the mean Nyquist frequency of 1.504221 cycle/m.

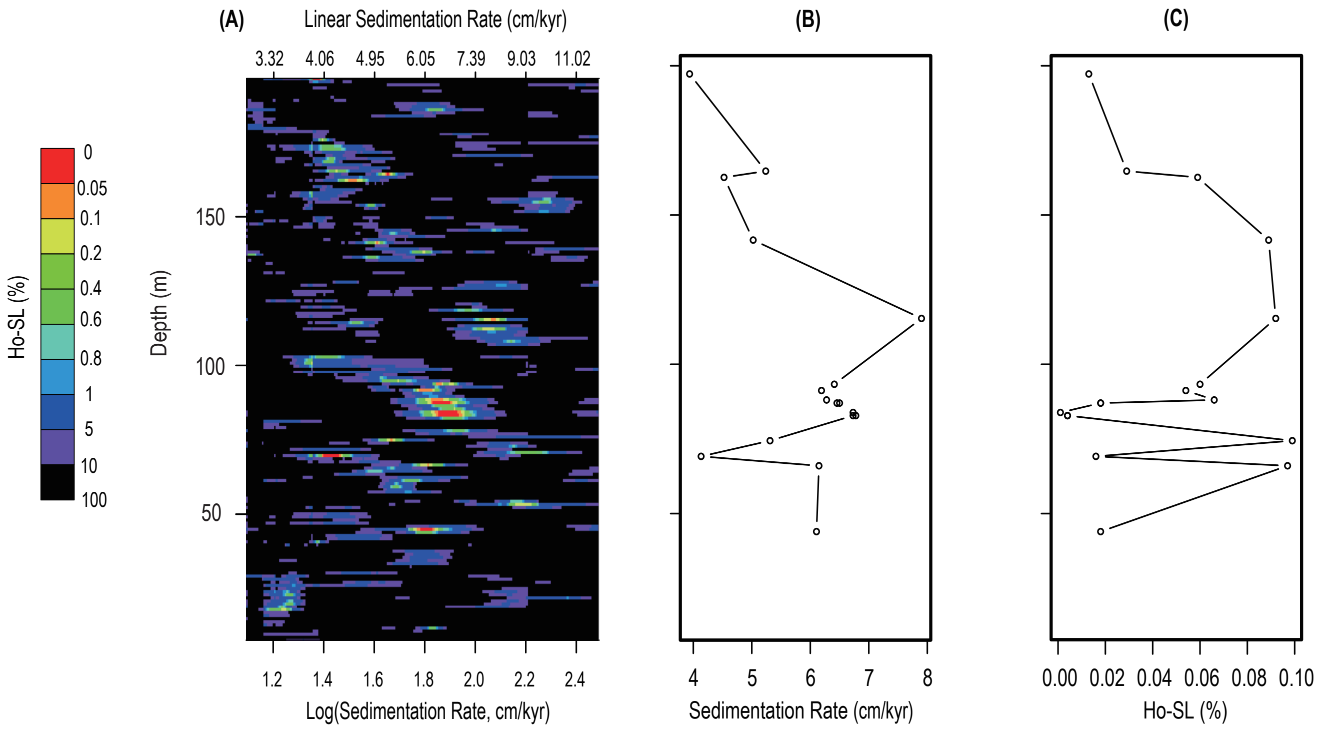

Figure S8. Astrochronologic testing of the Majiashan section using evolutive ASM analysis. (A) Evolutive ASM plot, displaying Ho-SL values for the significant harmonic F-test results in each 12-meter window of Figure S7D (90% confidence level), across sedimentation rates spanning 3 to 12 cm/kyr. (B and C) Summary of evolutive ASM results, using a threshold Ho-SL value of 0.1 to identify optimal sedimentation rates. Panel B displays each sedimentation rate, and panel C displays the associated Ho-SL. Detailed results are shown in Table S3.

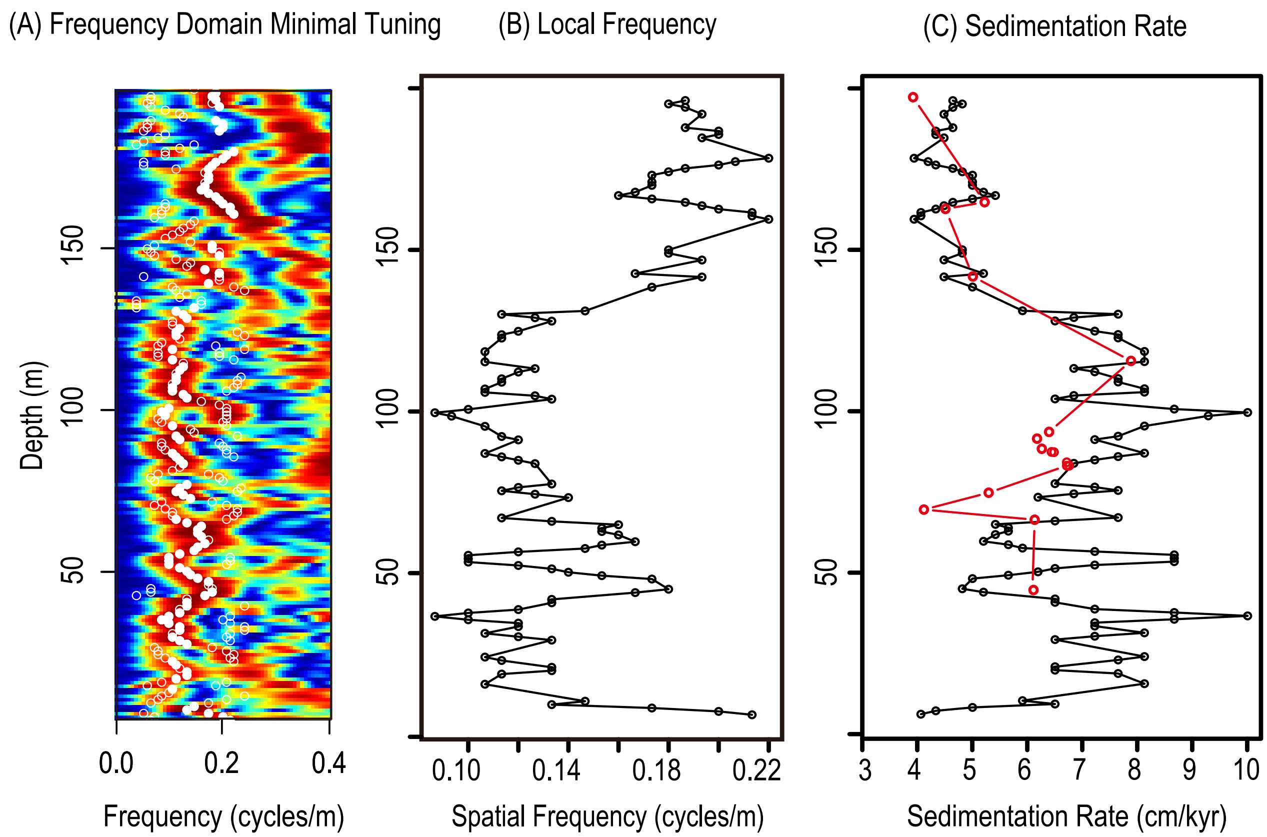

Figure S9. Frequency tracking for minimal tuning. The short-term eccentricity cyclicity (E2/2+E3/2=115.30 kyr) can be tracked in the EHA harmonic F-test confidence level results by setting the fmin=0.01 and fmax=0.25 based on the spatial frequencies calculated by EASM results. The EASM-derived optimal sedimentation rates are shown in red together with the calculated sedimentation rates based on spatial frequency tracking.

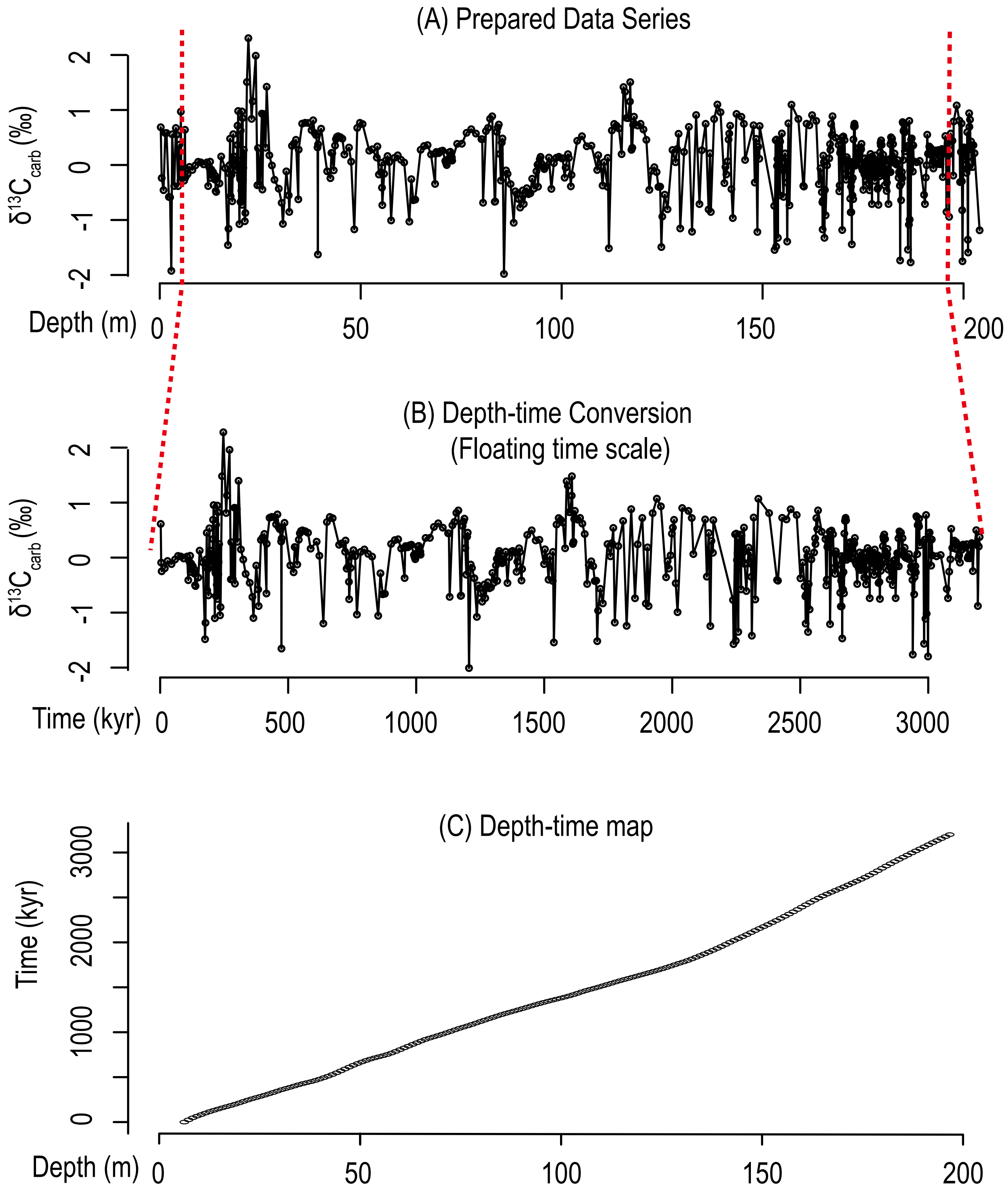

Figure S10. Tuned record and depth-time plot derived by frequency domain minimal tuning to the mean short eccentricity cycle. The red dotted lines show the correlated depth and time.

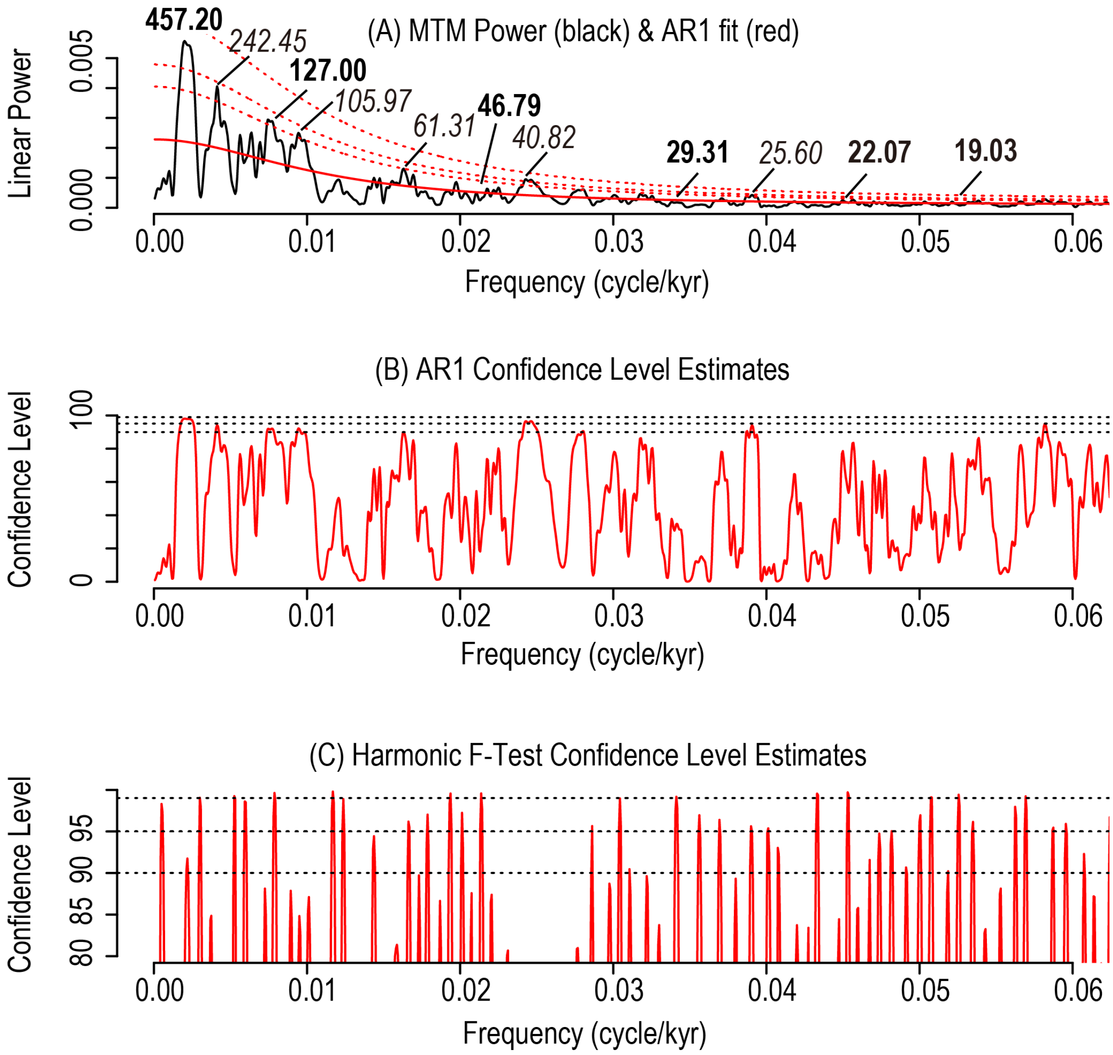

Figure S11. MTM results of the tuned data, using three tapers and a time-bandwidth product of 2. Note that the numbers in A represents the major periods in kyr. These peaks achieve the 90% confidence level for both the MTM harmonic F-test and the AR1 red noise model (bold) or the AR1 noise model only (italics).

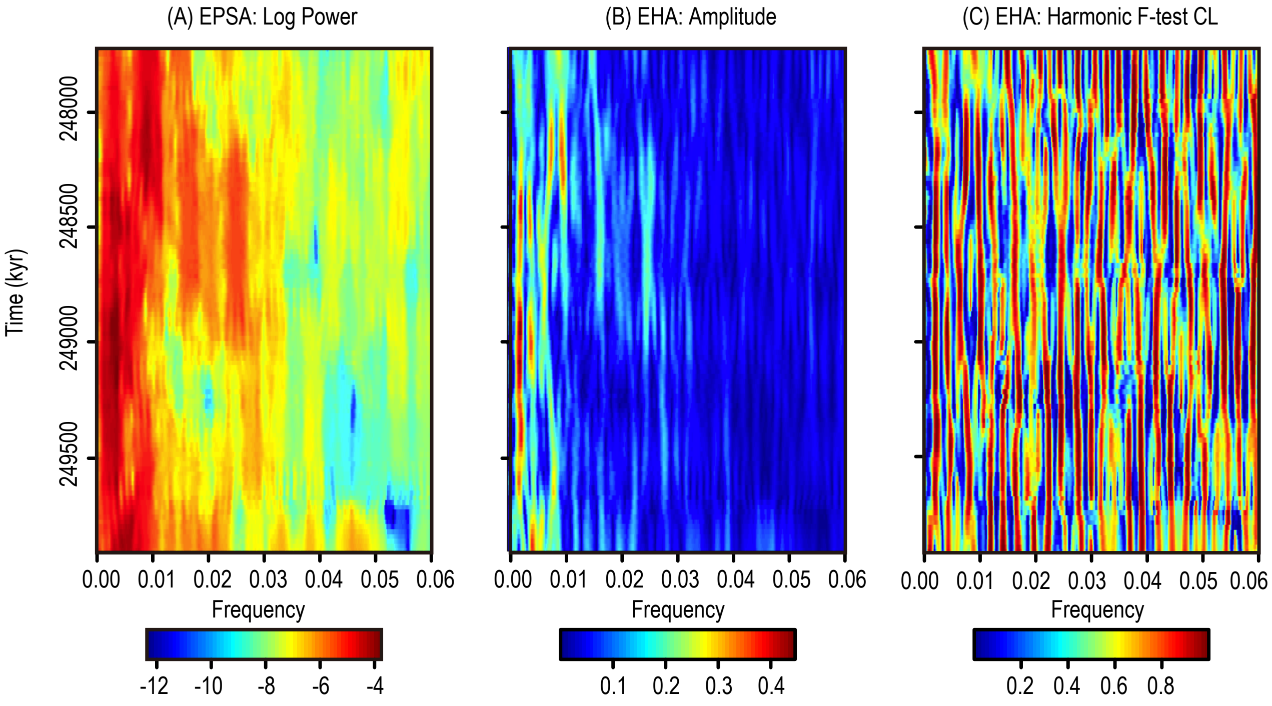

Figure S12. EHA of tuned data on time series, using a moving window of 1000 kyr, 3 tapers and a time-bandwidth product of 2.

Table S1 Majiashan $\delta^{13}C_{carb}$ data

(Note that the Depth 0 m marks the base of Helongshan Formation, 18.6 m below the Smithian-Spathian boundary fish-nodule shales)

Depth (m)	$\delta^{13}C_{PDB}$ (‰)
203.00	-3.9
202.73	-2.2
202.46	-2.0
202.24	-2.4
202.08	-2.0
201.97	-2.2
201.87	-1.8
201.74	-1.9
201.61	-1.4
201.46	-1.5
201.39	-1.2
201.28	-2.2
201.16	-1.8
201.14	-3.7
201.07	-3.4
200.93	-1.5
200.82	-2.1
200.74	-2.2
200.57	-2.0
200.37	-1.6
200.18	-1.6
199.93	-1.7
199.74	-3.6
199.66	-2.6
199.60	-2.1
199.51	-1.0
199.35	-1.5
199.14	-2.0
199.03	-3.7
198.96	-0.8
198.29	-0.4
197.66	-3.6
197.55	-0.5
197.45	-1.5
197.23	-0.8
196.81	-0.5
196.60	-0.6
196.51	-0.6
196.41	-1.9
196.24	-0.7
196.09	-0.5
195.93	-0.6
195.76	-1.6
195.64	-0.5
195.47	-0.2
195.20	-0.4
195.03	-0.4
194.84	-0.8
194.62	-0.3
194.43	-0.4
194.23	-0.5
194.02	-0.5
193.89	-0.5
193.73	-0.2
193.47	-0.3
193.31	-0.2
193.05	-0.2
192.81	-0.3
192.66	-0.3
192.50	-0.5
192.36	-0.4
192.22	-0.5
192.08	-0.3
191.93	-0.2
191.81	-0.2
191.68	-0.3
191.49	-0.2
191.15	-0.4
190.97	0.0
190.82	-0.6
190.61	-0.9
190.40	-1.4
190.18	-1.2
189.09	-0.8
188.34	-1.0
187.58	-1.1

187.49	-0.8
187.38	-1.3
187.26	-0.8
187.11	-0.7
186.95	-0.7
186.84	-3.0
186.72	-1.3
186.60	-2.2
186.50	-0.4
186.40	-2.3
186.28	-0.9
186.17	-2.8
186.05	-0.9
185.42	-1.0
185.32	-0.5
185.31	-0.7
185.19	-0.6
185.07	-0.7
185.00	-1.2
184.93	-0.5
184.84	-0.5
184.75	-1.5
184.67	-1.4
184.60	-1.5
184.53	-1.6
184.47	-1.9
184.36	-1.5
184.22	-3.0
184.16	-1.6
184.04	-1.3

183.93	-1.4
183.83	-1.5
183.58	-1.2
183.42	-1.3
183.27	-1.3
183.09	-1.3
182.93	-1.3
182.83	-1.1
182.71	-1.3
182.58	-1.3
182.47	-1.0
182.36	-1.2
182.21	-0.9
182.05	-0.9
181.95	-0.9
181.85	-1.5
181.79	-1.0
181.73	-1.3
181.59	-1.3
181.46	-1.6
181.33	-2.2
181.20	-1.5
181.05	-1.6
180.98	-1.6
180.92	-1.9
180.86	-2.0
180.75	-1.8
180.59	-1.7
180.48	-1.8
180.36	-2.0

180.25	-1.8
180.14	-1.9
180.01	-1.6
179.96	-1.7
179.89	-1.7
179.78	-1.7
179.63	-1.4
179.49	-1.4
179.42	-1.5
179.35	-1.4
179.31	-1.8
179.27	-1.6
179.19	-1.9
179.08	-2.0
178.96	-2.1
178.89	-2.0
178.82	-2.6
178.74	-2.3
178.61	-2.3
178.49	-1.9
178.44	-2.0
178.39	-2.0
178.34	-1.9
178.24	-1.9
178.10	-2.2
178.00	-1.8
177.89	-2.3
177.74	-2.0
177.67	-1.9
177.56	-2.1

177.40	-2.0
177.32	-2.0
177.27	-1.8
177.20	-2.7
177.12	-1.8
177.01	-1.9
176.85	-1.7
176.69	-1.8
176.59	-2.3
176.51	-2.1
176.42	-2.2
176.30	-2.2
176.23	-1.7
176.16	-1.8
176.07	-1.9
175.96	-1.8
175.72	-1.8
175.70	-1.8
175.63	-1.6
175.57	-1.6
175.49	-1.4
175.39	-1.6
175.33	-1.7
175.22	-1.5
175.06	-1.9
174.90	-1.4
174.75	-1.4
174.61	-0.9
174.51	-0.9
174.45	-0.9

174.37	-0.9
174.25	-0.9
174.12	-0.9
174.06	-1.1
174.00	-1.1
173.87	-1.0
173.74	-0.8
173.62	-1.0
173.50	-0.9
173.45	-1.1
173.38	-1.1
173.24	-1.2
173.06	-1.2
172.94	-0.5
172.86	-0.5
172.81	-0.6
172.74	-0.6
172.66	-1.2
172.61	-0.8
172.54	-1.9
172.44	-1.6
172.36	-1.9
172.33	-1.3
172.26	-1.4
172.20	-2.8
172.14	-1.6
172.08	-2.2
172.03	-1.3
172.00	-1.5
171.99	-1.7

171.97	-1.5
171.92	-2.2
171.81	-2.0
170.95	-1.0
170.86	-1.0
170.77	-1.4
170.60	-1.2
170.40	-0.6
170.10	-0.5
169.81	-2.1
169.73	-0.4
169.61	-0.5
169.56	-0.5
169.44	-0.6
169.27	-0.6
169.17	-1.2
169.09	-1.3
168.90	-0.4
168.15	-0.3
167.40	-0.1
167.23	-0.5
167.05	-0.3
166.87	-0.8
166.79	-0.7
166.59	-1.0
166.04	-1.1
165.68	-2.1
165.50	-1.6
165.39	-2.5
165.25	-1.1

165.11	-1.0
165.00	-1.8
164.94	-2.3
164.88	-1.9
164.74	-1.7
164.39	-1.3
163.99	-1.6
163.34	-0.4
162.32	-0.4
161.63	-0.6
160.94	-0.7
160.28	-1.9
160.14	-1.9
158.89	-0.8
157.19	-0.7
156.69	-2.6
156.42	-1.2
156.12	-3.4
156.03	-2.3
155.97	-2.1
155.90	-1.9
155.79	-2.1
155.58	-1.9
155.20	-2.7
154.90	-2.3
154.79	-1.6
154.69	-2.1
154.53	-2.3
154.37	-1.8
154.29	-2.8

154.18	-1.8
154.03	-2.0
153.91	-2.0
153.80	-3.6
153.69	-2.2
153.58	-2.3
153.45	-1.9
153.37	-3.8
153.26	-1.9
152.98	-3.9
152.89	-3.1
149.26	-1.6
149.18	-2.2
149.10	-5.0
148.91	-2.0
148.70	-3.5
148.40	-1.8
148.06	-2.6
147.92	-4.3
147.75	-1.5
145.64	-1.9
145.34	-1.2
144.73	-1.0
143.51	-0.8
142.59	-2.6
142.24	-2.1
141.93	-0.9
141.71	-1.0
141.53	-1.1
141.30	-1.5

140.99	-1.7
140.76	-1.5
140.49	-1.8
139.53	-0.5
138.75	-0.3
137.93	-0.6
137.11	-2.2
136.90	-1.2
136.56	-2.2
135.83	-0.6
134.86	-1.1
134.29	-2.1
133.46	-0.4
132.44	-2.6
131.65	-0.6
130.46	-1.1
129.51	-2.4
129.10	-0.7
128.72	-0.9
128.35	-1.1
127.59	-0.8
126.75	-4.3
126.34	-1.7
125.66	-1.4
125.05	-1.7
124.81	-2.2
124.51	-1.1
124.09	-1.0
123.63	-0.7
123.06	-0.5

122.31	-0.4
121.83	-0.6
121.08	0.4
120.36	0.7
119.64	0.9
118.92	0.9
117.99	1.2
117.78	1.1
117.58	0.7
117.37	0.7
117.07	2.0
116.99	1.6
116.54	1.3
116.16	1.4
115.78	1.9
115.40	2.0
114.92	0.8
114.24	1.3
113.66	1.4
113.07	1.4
112.49	1.2
111.74	-0.9
111.48	0.7
111.40	0.2
111.17	-2.6
111.05	-2.1
110.56	0.6
110.19	0.2
109.81	0.5
109.10	0.7

108.85	0.4
107.42	0.8
106.24	0.7
105.06	0.9
103.88	0.8
102.46	0.2
102.29	0.0
101.80	0.6
101.57	0.6
101.00	0.4
100.74	0.2
100.26	0.5
100.09	0.5
99.31	0.6
98.69	0.5
98.07	0.4
97.45	-0.1
96.83	0.5
95.94	0.3
95.68	0.2
95.42	0.3
94.96	0.3
94.76	0.5
94.56	0.4
94.32	0.3
94.12	-0.1
94.00	0.3
93.72	0.2
93.12	-0.1
92.67	0.1

92.23	-0.1
91.78	-0.1
91.33	0.1
90.57	-0.1
90.33	0.2
89.68	0.0
89.28	0.3
88.88	0.5
88.47	0.6
88.07	0.1
87.29	1.0
86.54	1.4
86.16	1.5
85.65	-0.2
85.28	2.3
85.10	2.3
84.89	1.7
84.61	2.2
84.36	2.8
84.11	2.9
83.86	2.8
83.46	1.7
83.35	1.7
82.72	3.4
82.20	3.5
81.68	3.5
81.16	3.5
80.44	2.3
80.24	3.5
80.04	3.5

78.67	3.8
77.50	3.9
76.34	3.9
75.17	3.8
74.00	3.9
72.76	3.6
72.68	3.7
72.60	3.7
72.28	3.8
72.05	3.8
71.82	3.6
71.41	3.5
71.23	3.5
71.05	3.6
70.39	3.7
69.91	3.7
69.43	3.6
68.80	3.6
68.65	3.6
68.50	3.0
67.50	3.5
66.64	3.6
65.78	3.5
64.92	3.3
63.57	2.3
63.08	2.3
62.59	2.6
62.10	1.8
60.82	2.8
60.02	2.8

59.22	2.8
58.42	2.7
57.56	1.5
57.10	2.6
56.68	2.7
56.26	2.6
55.84	2.3
55.37	1.7
55.29	2.1
55.21	2.5
55.03	2.3
54.53	2.3
54.15	2.8
54.01	2.6
52.87	2.8
52.13	2.8
51.26	-0.2
50.47	3.4
49.81	3.4
49.14	3.4
48.41	1.6
47.62	2.9
47.08	3.2
46.49	-2.1
45.94	3.2
45.44	3.5
44.94	3.6
44.37	3.7
44.08	3.7
43.85	3.7

43.62	3.6
43.29	3.2
42.92	3.5
42.72	3.6
42.46	3.1
41.65	3.3
40.15	4.2
39.40	4.0
39.35	2.0
39.26	3.9
38.88	4.2
38.53	4.2
38.18	4.5
37.76	4.3
36.94	4.5
36.19	4.5
35.43	4.5
34.56	4.1
34.47	3.2
33.89	4.2
33.40	4.2
32.90	4.1
32.22	2.9
32.08	3.2
31.50	3.6
30.64	2.6
30.34	3.0
29.61	3.2
28.55	3.3
27.91	3.5

27.27	3.6
26.60	4.9
26.31	3.8
26.11	4.3
25.82	4.3
25.54	2.9
25.40	4.2
24.92	2.8
24.44	3.4
24.30	2.7
23.88	4.9
23.12	3.8
22.85	3.3
22.55	-1.1
22.05	4.4
21.69	3.4
21.30	0.8
21.16	0.5
20.91	1.7
20.77	1.3
20.69	1.0
20.59	2.0
20.52	0.4
20.46	2.0
20.31	0.5
20.14	1.5
20.09	0.1
20.04	0.5
19.98	0.5
19.93	0.5

19.82	-0.5
19.64	0.4
19.55	0.5
19.50	1.3
19.41	0.2
19.35	1.0
19.30	0.6
19.12	0.0
18.93	0.0
18.75	-0.2
18.55	-0.3
18.46	-0.3
18.38	-0.4
18.30	0.0
18.03	-1.4
17.83	-1.0
17.63	-1.5
17.56	-1.1
17.49	-0.5
17.37	-1.1
17.21	-1.3
17.10	-2.4
16.97	-2.8
16.28	-1.7
15.29	-1.8
14.86	-2.4
14.03	-2.8
13.90	-2.8
13.86	-2.6
13.77	-2.5

13.74	-2.6
13.62	-2.6
13.26	-2.6
12.82	-2.6
12.68	-2.8
12.53	-2.5
12.31	-2.6
12.05	-3.0
11.73	-2.6
10.91	-2.7
10.23	-2.7
9.55	-2.8
8.87	-2.9
8.11	-3.0
7.40	-3.0
6.92	-3.1
6.42	-3.2
6.34	-3.1
6.26	-2.4
6.08	-3.3
5.68	-3.1
5.37	-3.3
5.25	-2.1
5.15	-3.4
4.88	-2.7
4.55	-3.5
4.19	-2.6
4.01	-2.5
3.61	-3.6
3.09	-2.7

2.88	-5.2		1.68	-2.7		0.48	-3.6
2.56	-3.8		1.38	-2.7		0.24	-2.7
2.27	-3.8		0.94	-3.8			

Table S2 Dominant Eccentricity, Obliquity and precession terms from La04 & La10 with harmonic confidence level above 90%

Frequency (cycle/kyr)	Period (kyr/cycle)	Harmonic_CL (%)
0.0025	400.00	99.99
0.0076	131.58	96.69
0.0101	99.01	99.24
0.0247 (weaker signal)	40.49	99.99
0.0305	32.79	99.60
0.0398 (weaker signal)	25.13	96.96
0.0483	20.70	99.44
0.0508	19.69	99.60
0.0586	17.06	99.88

Table S3 Ho-SL minima, the sedimentation rate and the observed calibrated periods in E-ASM

Depth (m)	Sedimentation rate (cm/kyr)	Ho-SL (%)	Period (kyr/cycle)
43.97	6.11	0.018	98.27
			36.67
			20.82
			16.16
66.02	6.15	0.097	121.99
			34.85
			20.68
			17.18
			12.77
69.17	4.13	0.016	106.74
			36.66
			19.94
74.42	5.31	0.099	148.64
			40.93
			33.62
			20.61
			16.32
82.82	6.73	0.004	123.81
			36.53
			18.73
83.87	6.73	0.001	117.29
			35.94
			18.73
87.02	6.50	0.018	144.22
			36.06
			18.91
88.07	6.28	0.066	170.67
			35.66
			19.58
91.22	6.19	0.054	134.60
			41.07
			31.47
			19.70
			16.94
			13.46
93.32	6.41	0.060	106.36
			38.36

			30.00
			20.53
			17.86
			13.22
115.37	7.90	0.092	118.66
			36.51
			18.80
			11.72
141.62	5.02	0.089	102.97
			58.55
			35.13
			20.59
			16.59
162.62	4.53	0.059	110.50
			39.00
			19.61
			17.63
164.72	5.24	0.029	102.28
			39.23
			34.09
			20.60
			16.46
			13.70
197.27	3.94	0.013	100.28
			43.30
			30.48
			20.27
			17.01

Table S4 ASM-derived sedimentation rates, floating timescale and calibrated anchored timescale
(Note that the floating timescale is "anchored" at 247.95 Ma with depth 162.62 m)

Depth (m)	Spatial frequency (cycle/m)	Sedimentation rate (cm/kyr)	Floating timescale (kyr)	Anchored timescale (kyr)
6.17	0.2133	4.07	0.00	250419.74
7.22	0.2000	4.34	25.02	250394.72
8.27	0.1733	5.00	47.62	250372.12
9.32	0.1333	6.50	66.18	250353.56
10.37	0.1467	5.91	83.13	250336.61
15.62	0.1067	8.13	158.58	250261.16
18.77	0.1133	7.65	198.51	250221.23
19.82	0.1333	6.50	213.44	250206.30
20.87	0.1333	6.50	229.58	250190.16
22.97	0.1133	7.65	259.34	250160.39
24.02	0.1067	8.13	272.66	250147.08
29.27	0.1333	6.50	344.73	250075.01
30.32	0.1200	7.23	360.06	250059.68
31.37	0.1067	8.13	373.78	250045.96
33.47	0.1200	7.23	401.18	250018.56
34.52	0.1200	7.23	415.70	250004.04
35.57	0.1000	8.67	429.02	249990.72
36.62	0.0867	10.01	440.32	249979.42
37.67	0.1000	8.67	451.62	249968.12
38.72	0.1200	7.23	464.94	249954.80
40.82	0.1333	6.50	495.56	249924.18
41.87	0.1333	6.50	511.71	249908.03
43.97	0.1667	5.20	547.80	249871.94
45.02	0.1800	4.82	568.79	249850.95
48.17	0.1733	5.00	632.94	249786.80
49.22	0.1533	5.66	652.71	249767.03
50.27	0.1400	6.20	670.47	249749.27
51.32	0.1333	6.50	687.01	249732.73
52.37	0.1200	7.23	702.35	249717.39
53.42	0.1000	8.67	715.66	249704.07
54.47	0.1000	8.67	727.77	249691.97
55.52	0.1000	8.67	739.88	249679.86
56.57	0.1200	7.23	753.19	249666.54
57.62	0.1467	5.91	769.34	249650.40
58.67	0.1533	5.66	787.50	249632.24

59.72	0.1667	5.20	806.87	249612.87
61.82	0.1600	5.42	846.41	249573.33
62.87	0.1533	5.66	865.37	249554.37
63.92	0.1533	5.66	883.94	249535.80
64.97	0.1600	5.42	902.90	249516.84
66.02	0.1333	6.50	920.66	249499.08
67.07	0.1133	7.65	935.59	249484.15
73.37	0.1400	6.20	1026.94	249392.80
74.42	0.1267	6.85	1043.08	249376.66
75.47	0.1133	7.65	1057.61	249362.13
76.52	0.1200	7.23	1071.73	249348.01
77.57	0.1333	6.50	1087.07	249332.67
83.87	0.1267	6.85	1181.46	249238.28
84.92	0.1200	7.23	1196.39	249223.35
85.97	0.1133	7.65	1210.51	249209.23
87.02	0.1067	8.13	1223.83	249195.91
91.22	0.1200	7.23	1278.59	249141.14
92.27	0.1133	7.65	1292.72	249127.02
95.42	0.1067	8.13	1332.65	249087.09
98.57	0.0933	9.29	1368.87	249050.87
99.62	0.0867	10.01	1379.77	249039.97
100.67	0.1000	8.67	1391.07	249028.67
103.82	0.1333	6.50	1432.93	248986.81
104.87	0.1267	6.85	1448.66	248971.07
105.92	0.1067	8.13	1462.79	248956.95
106.97	0.1067	8.13	1475.70	248944.04
109.07	0.1133	7.65	1502.32	248917.41
110.12	0.1133	7.65	1516.05	248903.69
112.22	0.1200	7.23	1544.28	248875.46
113.27	0.1267	6.85	1559.21	248860.53
115.37	0.1067	8.13	1587.36	248832.38
118.52	0.1067	8.13	1626.10	248793.64
122.72	0.1133	7.65	1679.34	248740.40
123.77	0.1133	7.65	1693.06	248726.68
124.82	0.1200	7.23	1707.18	248712.56
127.97	0.1333	6.50	1753.11	248666.63
129.02	0.1267	6.85	1768.85	248650.89
130.07	0.1133	7.65	1783.38	248636.36
131.12	0.1467	5.91	1799.12	248620.62
138.47	0.1733	5.00	1934.09	248485.65
141.62	0.1933	4.49	2000.56	248419.18

142.67	0.1667	5.20	2022.35	248397.39
146.87	0.1933	4.49	2109.22	248310.52
148.97	0.1800	4.82	2154.39	248265.35
150.02	0.1800	4.82	2176.18	248243.56
159.47	0.2200	3.94	2392.66	248027.08
160.52	0.2133	4.07	2418.89	248000.85
161.57	0.2133	4.07	2444.72	247975.02
162.62	0.2000	4.34	2469.74	247950.00
163.67	0.1933	4.49	2493.55	247926.19
164.72	0.1867	4.65	2516.55	247903.19
165.77	0.1733	5.00	2538.34	247881.40
166.82	0.1600	5.42	2558.52	247861.22
167.87	0.1667	5.20	2578.29	247841.45
169.97	0.1733	5.00	2619.45	247800.29
171.02	0.1733	5.00	2640.43	247779.31
173.12	0.1733	5.00	2682.40	247737.34
174.17	0.1800	4.82	2703.79	247715.95
175.22	0.1867	4.65	2725.99	247693.75
176.27	0.2000	4.34	2749.39	247670.35
177.32	0.2067	4.20	2774.01	247645.73
178.37	0.2200	3.94	2799.83	247619.90
184.67	0.1933	4.49	2949.55	247470.19
185.72	0.2000	4.34	2973.36	247446.38
186.77	0.2000	4.34	2997.57	247422.17
187.82	0.1867	4.65	3020.98	247398.76
192.02	0.1933	4.49	3112.97	247306.77
194.12	0.1867	4.65	3158.97	247260.77
195.17	0.1800	4.82	3181.16	247238.58
196.22	0.1867	4.65	3203.36	247216.38

Table S5 The calibrated periods of 115.30-kyr-tuned time series with harmonic confidence level above 90%

Frequency (cycle/kyr)	Period (kyr/cycle)	Harmonic_CL (%)
0.0005	2000.25	98.3085
0.0022	457.20	91.7422
0.0030	333.38	99.0537
0.0052	190.50	99.2708
0.0059	168.44	98.6214
0.0079	127.00	99.6306
0.0117	85.57	99.7944
0.0124	80.82	98.8901
0.0144	69.57	94.4308
0.0166	60.16	96.1840
0.0179	55.95	97.0252
0.0194	51.62	99.5640
0.0201	49.70	97.2414
0.0214	46.79	99.5757
0.0286	34.94	95.6457
0.0304	32.86	98.9771
0.0311	32.20	90.4614
0.0341	29.31	99.1727
0.0356	28.07	96.9526
0.0369	27.08	96.3969
0.0390	25.64	95.6264
0.0401	24.93	95.3727
0.0407	24.54	93.0221
0.0433	23.09	99.5681
0.0453	22.07	99.7282
0.0467	21.39	91.5733
0.0474	21.11	94.7451
0.0482	20.75	95.0328
0.0491	20.36	90.6702
0.0501	19.98	96.9484
0.0507	19.71	99.1152
0.0519	19.28	90.2115
0.0526	19.03	99.4204
0.0535	18.69	96.1510
0.0562	17.78	97.9624
0.0569	17.57	99.2255
0.0587	17.02	95.4832

0.0596	16.79	95.9078
0.0607	16.46	92.2948
0.0624	16.02	96.7175
0.0636	15.72	93.5093
0.0642	15.57	97.6864
0.0652	15.33	98.8311
0.0659	15.18	96.2150
0.0664	15.07	96.2683
0.0670	14.93	99.0760
0.0676	14.79	92.9160
0.0686	14.59	99.8355
0.0692	14.46	90.3791

References

1. Motani, R. *et al*. A basal ichthyosauriform with a short snout from the Lower Triassic of China. *Nature* **517**, 485-488 (2015).
2. Jiang, D. Y. *et al*. The Early Triassic eosauropterygian Majiashanosaurus discocoracoidis, gen. et sp. nov.(Reptilia, Sauropterygia), from Chaohu, Anhui Province, People's Republic of China. *Journal of Vertebrate Paleontology* **34**, 1044-1052 (2014).
3. Tong, J. & H. Yin. The Lower Triassic of South China. *Journal of Asian Earth Sciences* **20**, 803-815 (2002).
4. Sun, Z. *et al*. Magnetostratigraphy of the Lower Triassic beds from Chaohu (China) and its implications for the Induan–Olenekian stage boundary. *Earth and Planetary Science Letters* **279**, 350-361 (2009).
5. Li, S. Y. *et al.* The Lower Triassic cyclic deposition in Chaohu, Anhui Province, China. *Palaeogeography, Palaeoclimatology, Palaeoecology* **252**, 188-19 (2007).
6. Zhao, L. S. *et al*. Lower Triassic conodont sequence in Chaohu, Anhui Province, China and its global correlation. *Palaeogeography, Palaeoclimatology, Palaeoecology* **252**, 24-38 (2007).
7. Tong, J. & Y. D. Zakharov. Lower Triassic ammonoid zonation in Chaohu, Anhui Province, China. *Albertiana* **2004**, 65−69 (2004)
8. Tong, J. *et al*. Early Triassic carbon isotope excursions from South China: proxies for devastation and restoration of marine ecosystems following the end-Permian mass extinction. *Geological Journal* **42**, 371-389 (2007).
9. Song, H. *et al*. Large vertical $\delta^{13}C_{DIC}$ gradients in Early Triassic seas of the South China craton: Implications for oceanographic changes related to Siberian Traps volcanism. *Global and Planetary Change* **105**, 7-20 (2013).
10. Giorgioni, M. *et al*. Orbital control on carbon cycle and oceanography in the mid-Cretaceous greenhouse. *Paleoceanography* **27**, PA1204 (2012).
11. Meyer, K. M. *et al*. Constraints on Early Triassic carbon cycle dynamics from paired organic and inorganic carbon isotope records. *Earth and Planetary Science Letters* **361**, 429-435 (2013).

12. Sun, Y. *et al*. Lethally Hot Temperatures During the Early Triassic Greenhouse. *Science* **338**, 366-370 (2012).
13. R Core Team. R: A language and environment for statistical computing. R Foundation for Statistical Computing, Vienna, Austria. URL http://www.R-project.org/ (2013).
14. Ovtcharova, M. *et al*. New Early to Middle Triassic U–Pb ages from South China: Calibration with ammonoid biochronozones and implications for the timing of the Triassic biotic recovery. *Earth and Planetary Science Letters* **243**, 463-475 (2006).
15. Lehrmann, D. J. *et al*. Timing of recovery from the end-Permian extinction: Geochronologic and biostratigraphic constraints from south China. *Geology* **34**, 1053-1056. (2006).
16. Thomson, D. J. Spectrum estimation and harmonic analysis. *Proceedings of the IEEE* **70**, 1055-1096 (1982).
17. Meyers, S. R. & B. B. Sageman Quantification of deep-time orbital forcing by average spectral misfit. *American Journal of Science* **307**, 773-792 (2007).
18. Meyers, S.R. Seeing Red in Cyclic Stratigraphy: Spectral Noise Estimation for Astrochronology. *Paleoceanography*, 27: PA3228 (2012).
19. Meyers, S.R. *et al*. Obliquity forcing of organic matter accumulation during Oceanic Anoxic Event 2. *Paleoceanography*, 27: PA3212. (2012).
20. Laskar, J. *et al.* A long-term numerical solution for the insolation quantities of the Earth. *Astronomy and Astrophysics* 428:261-285 (2004).
21. Laskar, J., Fienga, A., Gastineau, M. & Manche, H. La2010: A new orbital solution for the long-term motion of the Earth. *Astronomy and Astrophysics*. 532: A89 (2011).
22. Meyers, S. R. *et al*. Integrated Quantitative Stratigraphy of the Cenomanian-Turonian Bridge Creek Limestone Member Using Evolutive Harmonic Analysis and Stratigraphic Modeling. *Journal of Sedimentary Research* **71**, 628-644 (2001).
23. Scheyer, T. M. *et al*. Early Triassic Marine Biotic Recovery: The Predators' Perspective. *PLoS ONE* **9**(3), 1-20 (2014).

R_analysis

```
#######################################
# (1) LOAD THE R-PACKAGE 'ASTROCHRON'
#######################################
library(astrochron)

#######################################
# (2) READ DATA FILE
#######################################
# Read the carbon isotope data from file 'CarbonIso.csv'
dat<- read()

##########################
# (3) PREPARE TIME SERIES
##########################
# Remove the long-term trend with a Lowess smoother
c13_detrend<- noLow(dat,smooth=0.08)
# Remove carbon isotope data outliers
c13_trim<- trimAT(c13_detrend,thresh=-2,dir=1)
# The median sampling interval of the prepared data is 0.19 m, and the mean
sampling interval is 0.332398 m
# Place the carbon isotope data on a 15 cm sampling grid, using piecewise linear
interpolation. This
# sampling grid is appropriate for the MTM Harmonic F-test (overinterpolation is
not a problem).
c13<- linterp(c13_trim,dt=0.15)

###########################################
# (4) PERFORM EVOLUTIVE HARMONIC ANALYSIS
###########################################
# Use a 12 meter window, with five 3pi DPSS tapers.
#  * Search up to the mean Nyquist frequency of 1.504221 cycle/m
#  * Output F-test confidence level estimates for evolutive average spectral misfit
(ASM) analysis.
prob=eha(c13,win=12,step=1,tbw=3,pad=1000,fmax=1.504221,output=4,genplot=
4,pl=2)

######################################################################
# (5) IDENTIFY TARGET PERIODS FOR AVERAGE SPECTRAL MISFIT
ANALYSIS
######################################################################
# Obliquity and precession terms from Laskar et al. (2004)
model=etp(tmin=248000,tmax=249000,eWt=0)
eha(model,win=1000,fmax=0.1,sigID=T,pad=10000)
# eccentricity terms from Laskar et al. (2011)
```

```
model=getLaskar("la10d")
# Investigate a slightly larger interval for better estimates of the eccentricity periods
model=iso(model,xmin=247000,xmax=249000)
eha(model,win=2000,fmax=0.1,sigID=T,pad=20000)

##################################################
# (6) EVOLUTIVE AVERAGE SPECTRAL MISFIT ANALYSIS
##################################################
# Set up analysis parameters:
#  * Astronomical target frequencies are determined from Laskar et al. (2004) and
Laskar et al. (2011)
target1=c(1/400,1/131.58,1/99.01,1/40.49,1/32.79,1/20.70,1/19.69,1/17.06)
rayleigh=0.08333333
#  * Use average sampling interval to estimate the Nyquist frequency
nyquist=1.504221
#  * Longterm average sedimentation rates are constrained by three U/Pb ages of
Spathian in South China
#    from Ovtcharova et al. (2006) and Lehrmann et al. (2006).
#    Chin-10: 250.55 +/- 0.51 Ma (95% CI) for the earliest Spathian
#    PGD Tuff-1: 247.38 +/- 0.10 Ma (2 sigma) for the lastest Spathian
#    PGD Tuff-2: 247.32 +/- 0.08 Ma (2 sigma) for the lastest Spathian
#    The total duration between the youngest and oldest age of Spathian (combining
errors in
#    quadrature at 2 sigma) is 3.23 Ma +/- 0.53 Ma.
#    Given the range of plauisble correlation horizons into the Majiashan section,
#    the total stratigraphic thickness can range from 180 m to 190 m.  This indicates
#    longterm average sedimentation rates of 4.79 cm/kyr to 7.04 cm/kyr.
# Execute evolutive ASM analysis. This will take 10-20 minutes to complete.
res1=eAsm(prob,target=target1,rayleigh=rayleigh,nyquist=nyquist,sedmin=3,sedm
ax=12,numsed=200,siglevel=0.8,iter=100000,output=4)
# Track Ho-SL minima from evolutive ASM results
#  * Identify those results with Ho-SL less than or equal to 0.1%
pl(1); eAsmTrack(res1[1],threshold=0.1,ydir=1)

############################################################
# (7) EXAMINE SELECTED SPECTRA AND ASM-CALIBRATED PERIODS
############################################################

# F-test CL spectrum from 43.965 m
prob_43.965=extract(prob,get=43.965)
# Calculate calibrated periods in kyr (observed)
1/(peak(prob_43.965,level=0.8)[2]*0.06105410)

# F-test CL spectrum from 66.015 m
prob_66.015=extract(prob,get=66.015)
# Calculate calibrated periods in kyr (observed)
```

```
1/(peak(prob_66.015,level=0.8)[2]*0.06148090)

# F-test CL spectrum from 69.165 m
prob_69.165=extract(prob,get=69.165)
1/(peak(prob_69.165,level=0.8)[2]*0.04133243)

# F-test CL spectrum from 74.415 m
prob_74.415=extract(prob,get=74.415)
1/(peak(prob_74.415,level=0.8)[2]*0.05311367)

# F-test CL spectrum from 82.815 m
prob_82.815=extract(prob,get=82.815)
1/(peak(prob_82.815,level=0.8)[2]*0.06730864)

# F-test CL spectrum from 83.865 m
prob_83.865=extract(prob,get=83.865)
1/(peak(prob_83.865,level=0.8)[2]*0.06730864)

# F-test CL spectrum from 87.015 m
prob_87.015=extract(prob,get=87.015)
1/(peak(prob_87.015,level=0.8)[2]*0.06500454)

# F-test CL spectrum from 88.065 m
prob_88.065=extract(prob,get=88.065)
1/(peak(prob_88.065,level=0.8)[2]*0.06277931)

# F-test CL spectrum from 91.215 m
prob_91.215=extract(prob,get=91.215)
1/(peak(prob_91.215,level=0.8)[2]*0.06191069)

# F-test CL spectrum from 93.315 m
prob_93.315=extract(prob,get=93.315)
1/(peak(prob_93.315,level=0.8)[2]*0.06410513)

# F-test CL spectrum from 115.365 m
prob_115.365=extract(prob,get=115.365)
1/(peak(prob_115.365,level=0.8)[2]*0.07900519)

# F-test CL spectrum from 141.615 m
prob_141.615=extract(prob,get=141.615)
1/(peak(prob_141.615,level=0.8)[2]*0.05023459)

# F-test CL spectrum from 162.615 m
prob_162.615=extract(prob,get=162.615)
1/(peak(prob_162.615,level=0.8)[2]*0.04525030)
```

```
# F-test CL spectrum from 164.715 m
prob_164.715=extract(prob,get=164.715)
1/(peak(prob_164.715,level=0.8)[2]*0.05237879)

# F-test CL spectrum from 197.265 m
prob_197.265=extract(prob,get=197.265)
1/(peak(prob_197.265,level=0.8)[2]*0.03936524)

##############################################################
# (8) ASTRONOMICALLY-TUNE CARBON ISOTOPE DATA USING
#     FREQUENCY-DOMAIN MINIMAL TUNING (Meyers et al., 2001)
##############################################################
# Track short-term eccentricity in EHA harmonic F-test confidence level given the ASM-calibrated periods
# Track the average of E2 and E3 term on the basis of the ASM calibrated sedmentation rates
# Note that the Rayleigh frequency is 0.08333333 cycles/m
freqs=trackFreq(prob,fmin=0.01,fmax=0.25)

# convert spatial frequencies to sedimentation rates using average period of 115.30 kyr
sedrate=freq2sedrate(freqs,period=115.30)

# View the calibrated sedimentation rates on depth
sedrate

# Integrate the sedimentation rate curve to create a time-space map
time=sedrate2time(sedrate)

# View the calibrated time series
time

# The duration of specific interval can be calculated by the output of sedrate and time
# Tune the carbon isotope series using the time-space map
tuned=tune(c13_trim,time)

##################################################
# (9) PREPARE TUNED SERIES AND EVALUATE SPECTRA
##################################################
# Interpolate the tuned series. Median sampling interval is 3.497833 kyr and mean is 5.752486 kyr.
# Will use AR1 test; use a conservative interpolation to avoid introducing serial correlation.
datatuned=linterp(tuned, dt=7)
```

```
# Perform MTM analysis on the tuned series
spec=mtm(datatuned,tbw=2,pl=2,siglevel=0.9,xmax=0.06,output=1)

# identify periods of AR1 CL peaks that acheive the 90% AR1 CL
1/peak(cb(spec,c(1,4)),level=90)[2]

# Perform EHA on the tuned series
pwr=eha(datatuned,win=1000,step=20,pad=1000,fmax=0.06,output=2,ydir=-1)
plotEha(pwr,pl=1,ydir=-1)

###########################################################################
# (10) BANDPASS FILTERING AND ECCENTRICITY AMPLITUDE MODULATION ANALYSIS
###########################################################################
# Perform bandpass-filtering on the tuned series to extract long eccentricity (E1)
e1_data=bandpass(datatuned,flow=0.0015,fhigh=0.0028,xmax=0.02)

# Now extract short eccentricity (E2+E3)
e23_data=bandpass(datatuned,flow=0.006,fhigh=0.011,xmax=0.02)

# Perform bandpass-filtering on the eccentricity terms from Laskar et al. (2011)
model=getLaskar("la10d")
model=iso(model,xmin=247000,xmax=249000)
e1_model=bandpass(model,flow=0.0015,fhigh=0.0028,xmax=0.02) #long-term eccentricity
e23_model=bandpass(model,flow=0.006,fhigh=0.011,xmax=0.02) #short-term eccentricity

# Evaluate the alignment between the amplitude envelope of the filtered short-term eccentricity and the filtered long-term eccentricity
am_data=hilbert(bandpass(datatuned,flow=0.006,fhigh=0.011))

pl(1)
plot(s(am_data),type="l",ylim=c(-3,3))
lines(s(e1_data),col="red")
```
